# The Microtubule‐Associated Protein CsTON2 Interacts With CsTRM5 and CsSUN to Regulate Fruit Shape Development in Cucumber

**DOI:** 10.1111/pbi.70519

**Published:** 2025-12-29

**Authors:** Min Li, Xiaoli Li, Yuting He, Chuang Li, Chaoheng Gu, Chengzhen Sun, Xiao Ma, Yan Geng, Siyu Hu, Lijie Han, Liu Liu, Ye Liu, Zhihan Liu, Daixi She, Zhaoyang Zhou, Xiaofei Song, Yupeng Pan, Liying Yan, Xiaolan Zhang, Jianyu Zhao

**Affiliations:** ^1^ Beijing Key Laboratory of Growth and Developmental Regulation for Protected Vegetable Crops, Department of Vegetable Sciences China Agricultural University Beijing China; ^2^ Hebei Key Laboratory of Horticultural Germplasm Excavation and Innovative Utilization Hebei Normal University of Science & Technology Qinhuangdao China; ^3^ College of Horticulture Northwest A&F University Yangling China

**Keywords:** cell division, CsSUN, CsTON2, CsTRM5, fruit shape

## Abstract

Fruit shape is an important external quality trait that directly determines the market value. Modification of fruit shape has emerged as a key focus in crop improvement, but the regulatory network of fruit shape specifications remains largely unknown. Here, we identified a short fruit mutant (*sf5*) that was caused by a C‐to‐T single nucleotide polymorphism (SNP) in *TONNEAU2* (*CsTON2*), a microtubule‐associated gene encoding the B subunit of protein phosphatase 2A (PP2A). Overexpression of *CsTON2* in the *sf5* background partially rescued the mutant phenotype, while knockout of *CsTON2* led to severe developmental defects and dwarfism. We further demonstrated that CsTON2 physically interacts with CsTRM5 and CsSUN, two key regulators of fruit shape in cucumber. The SNP change of CsTON2 in *sf5* mutant impairs the interaction with CsTRM5 and CsSUN, and decreases the protein stability of CsSUN. Genetic analyses revealed that CsTON2, CsTRM5 and CsSUN coordinately regulate fruit shape development by modulating cell division direction in cucumber. Therefore, our findings shed insights into the role of microtubule‐associated protein complex in fruit shape determination and provide new gene targets for breeding cucumber varieties with favourable fruit shapes.

## 1 Introduction

Fruits represent one of the most significant hallmarks during angiosperms evolution that play important roles in plant reproduction and human diet (Doebley et al. 2006; Liu and Franks 2015). Fruit shape is an important external quality trait that directly determines the marketing value. The enormous phenotypic diversity of fruit shape in agricultural crops drives its modification as a key focus in crop enhancement (Li et al. 2023).

Among the diverse fruit types found in agricultural crops, cucumber ( *Cucumis sativus* L.) produces fleshy pepo fruit with dramatic variations in fruit shape and emerged as an ideal model for studying the genetic and molecular basis of fruit development (Pan et al. 2020; Zhang et al. 2025). The cucumber fruit is usually harvested immaturely (1–2 weeks after anthesis) (Wu et al. 2014) and the fruit shape is mainly determined by indicators such as fruit length (FL), fruit diameter (FD) and fruit shape index (FSI, the ratio of FL to FD) (Weng et al. 2015). After fruit initiation, the development of cucumber fruit is typically categorised into three distinct stages: the cell division phase (0–4 days after anthesis, DAA), the exponential expansion phase (4–12 DAA) and the post expansion growth phase (> 12 DAA) (Pan et al. 2020; Xie et al. 2023). These distinct developmental phases reflect the precise spatiotemporal coordination between cell division and expansion processes, which ultimately specify the final fruit shape (Périn et al. 2002; Marguerit et al. 2009; Zhou et al. 2020; Boualem et al. 2022).

Several transcription factors have been identified to regulate fruit shape in cucumber. *CsFUL1* ^*A*^ is a functional allele of the MADS‐box transcription factor *FRUITFULL* that negatively regulates fruit length by inhibiting cell division and expansion (Zhao et al. 2019). The YABBY transcription factor *CRABS CLAW* (*CRC*) is the causal gene of the major‐effect QTL *FS5.2* (*CsCRC*) that promotes fruit elongation by activating its target gene, auxin response protein (*CsARP1*), thereby stimulating cell expansion (Che et al. 2023). Recently, natural variations of *SEPALLATA2* (*SEP2*) in cucumber were shown to modulate fruit length through differential inhibitory effect on *CsCRC*‐mediated cell expansion (Song et al. 2024). Further, four genes affecting cucumber fruit length have been identified via map‐based cloning of EMS‐induced mutants, including *SF1* (*Short Fruit 1*), which encodes a cucurbit‐specific RING‐type E3 ubiquitin ligase; *SF2* (*Short Fruit 2*), which encodes a histone deacetylase complex 1 (HDC1) protein; *SF3* (*Short Fruit 3*), which encodes a subunit of microtubule‐severing enzyme katanin p60; and *SF4* (*Short Fruit 4*), which encodes a GlcNAc transferase (Xin et al. 2019; Zhang, Wang, et al. 2020; Wang et al. 2021; Zhang et al. 2023). QTL analysis in cucumber identified two interacting loci, *FS1.2* and *FS2.1*, which encode *CsSUN* and *TONNEAU1 RECRUITING MOTIF 5* (*CsTRM5*), respectively, contributing to fruit shape index (FSI) by regulating the direction of cell division. Loss of function in *CsSUN* or *CsTRM5* results in shorter and wider fruits in cucumber (Pan et al. 2017; Wu et al. 2018; Xie et al. 2023; Zhao et al. 2024). SUN encodes a protein belonging to the IQD family, which regulates organ shape through interactions with microtubules (Lazzaro et al. 2018). In tomato, the SUN protein localizes to microtubules and likely regulates cell number and fruit shape by altering microtubule structure during cell division (Xiao et al. 2008; Bao et al. 2021). SlIQD21a interacts with microtubule‐associated SlMAP70 proteins and regulates fruit elongation by manipulating microtubule function in tomato (Bao et al. 2023). On the other hand, CsTRM5 is a member of TRM family proteins that is crucial for assembling the TTP (TON1‐TRM‐PP2A) complex to demarcate the cortical plane of cell division. TRM proteins generally localize to microtubules where they recruit TON1 (TONNEAU1) through direct interaction (Drevensek et al. 2012). TRM proteins also recruit Protein Phosphatase 2A (PP2A) to microtubules to facilitate the formation of the preprophase band (PPB) during cell division (Spinner et al. 2010; Spinner et al. 2013; Schaefer et al. 2017; Wu et al. 2018). However, whether the regulatory process of CsTRM5 and CsSUN requires coordination with other proteins remains mysterious.

PP2A, a crucial member of the protein phosphatase family, is involved in plant development and stress responses in eukaryotic cells (Zhou et al. 2004; Blakeslee et al. 2008; Tseng and Briggs 2010; Durian et al. 2016; Li et al. 2019). PP2A forms a heterotrimeric complex consisting of a catalytic C subunit, a scaffolding A subunit, and a variable regulatory B subunit that determines the substrate selectivity and subcellular localization of the enzyme (Spinner et al. 2013). *TON2* encodes a regulatory subunit of PP2A that may participate in phosphorylation cascades to control the dynamic organisation of the cortical cytoskeleton in plant cells (Camilleri et al. 2002; Kirik et al. 2012). Previous data showed that *ton2* mutant plants display cell morphogenesis defects and *ton2* trichomes fail to develop branches (Traas et al. 1995; McClinton and Sung 1997). A total of 16 independent alleles of the *ton2* mutation were identified in *Arabidopsis*. Loss of function mutants such as *ton2‐5* and *ton2‐13* exhibit extreme phenotypes with a highly compressed apical‐basal axis, whereas *ton2‐12* and *ton2‐14* showed milder phenotypes with less pronounced morphological alterations (Camilleri et al. 2002; Kirik et al. 2012). *GRAIN WIDTH7* (*GW7*) encodes a rice homologue of the Arabidopsis TRM protein, which interacts with OsTON2 and promotes longitudinal cell elongation, thus gives rise to long and narrow leaf blades (Lee et al. 2006; Wang et al. 2015; Wu et al. 2023). OFPs modulate cell elongation by directly interacting with TON2, thus affecting microtubule reorientation and influencing the longitudinal expansion of cotyledon pavement cells (Zhang, Wu, et al. 2020). However, whether and how TON2 participates in fruit shape specification through interaction with microtubule‐associated proteins remains largely unknown.

In this study, we isolated a short and wide fruit mutant, *sf5*. Genetic analysis identified that *CsTON2* is the causal gene underlying the *sf5* mutant phenotype. We further demonstrated that CsTON2 directly interacts with CsTRM5 and CsSUN at the protein level. The SNP change of CsTON2 in the *sf5* mutant weakens the interaction with CsTRM5 and CsSUN, and thus decreases the protein stability of CsSUN. Genetic analyses showed that CsTON2, CsTRM5 and CsSUN coordinately regulate fruit shape formation by modulating cell division direction in cucumber.

## 2 Materials and Methods

### 2.1 Plant Materials and Growth Conditions

Cucumber ( *Cucumis sativus* L.) inbred lines XTMC and R1461 were used in this study for genetic transformation and expression analysis. Seeds were soaked and germinated in the dark at 28°C, then transferred to a growth chamber (16 h of light/8 h of darkness) for cultivation. After the two cotyledons were fully expanded, they were transplanted to the greenhouse of China Agricultural University, where standard water, fertiliser management and pest control measures were adopted. *N. bethamiana* was planted in a growth chamber at 24°C with 16 h of light/8 h of darkness. Plants about 5 weeks old were used for protein interaction assays and subcellular localization analysis.

### 2.2 Gene Mapping

Among the 30 strains from the (WT × *sf5*) F_2_ population that exhibited the short fruit mutation phenotype, the DNA of the female flower buds was extracted and then batch sequencing was performed (Biomarker Biotechnology Co. Ltd., Beijing). According to the previously described method (Abe et al. 2012), the wild‐type 6457 genome was used as the reference genomic sequence and MutMap analysis was conducted. The candidate genes are listed in DataSet S1.

### 2.3 Subcellular Localization

The full‐length CDS of *CsTON2* and *CsTON2* ^*m*^ were amplified from the WT and *sf5,* and were introduced into the vector pCAMBIA1300‐GFP to generate the fusion vectors CsTON2‐GFP and CsTON2^m^‐GFP. The CDS of *CsSUN* was cloned and introduced into the vector pCAMBIA1300‐GFP and pCAMBIA1300‐mcherry to generate the fusion vector CsSUN‐GFP and CsSUN‐mcherry. MBD‐mCherry as a microtubule marker and CsSWEET7a‐mCherry as a cell membrane marker (a previously reported cell membrane‐localized protein in cucumber) (Li et al. 2021). *N. benthamiana* leaves that harboured a nuclear marker (nuclear‐mCherry) were used to show that CsTON2‐GFP and CsTON2^m^‐GFP localize to the nucleus. Subsequently, these constructs were transformed, respectively into *N. benthamiana* leaves. After being incubated in the dark for 24 h and then cultivated under light for 48 h, the epidermal cells of *N. benthamiana* leaves were observed using a confocal laser scanning microscope (ZEISS/LSM880).

### 2.4 Phylogenetic Analysis

To identify homologous proteins of CsTON2, the amino acid sequences of the TON2 from Arabidopsis were employed as queries to perform a BLASTp search against the NCBI database. For multiple sequence alignment of the full‐length amino acid sequences of all identified proteins, the Muscle 3.6 program (available at https://www.drive5.com/muscle/) was utilised. The resulting alignment was subsequently refined manually using GeneDoc 3.2. Phylogenetic analysis was conducted in MEGA6.0. Specifically, amino acid sequences were aligned with ClusterW and a maximum likelihood‐based phylogenetic tree was constructed with 1000 bootstrap replicates to assess node support. The gene information is listed in Table S2.

### 2.5 Cucumber Transformation

In order to obtain CRISPR/Cas9 gene‐edited plants targeting *CsTON2*, we designed and selected specific sgRNA target sites using the CRISPR‐P 2.0 website (https://crispr.hzau.edu.cn/CRISPR2/). Using the vector pCBC‐DT1T2 as a template, we generated PCR fragments containing two sgRNA target sites by amplification and then inserted them into the vector pKSE402 at the BsaI site using T4 ligase. The pKSE402 vector contains a green fluorescent protein reporter gene (Xing et al. 2014). To obtain the complementation transgenic line of *CsTON2*, the full‐length CDS of *CsTON2* was constructed into the intermediate vector pUC19‐Flag and the operation was carried out using the KpnI and BstBI sites, thereby generating the pUC19‐CsTON2‐Flag construct. Subsequently, the CsTON2‐Flag fragment was amplified using the recombinant vector as a template by PCR and it was ligated to the pCAMBIA1305 vector using the BamHI and BstEII sites, thereby generating the vector *pro35S*:CsTON2‐Flag (The *pro35S*:CsSUN‐Flag vector was obtained using the same method). A 2000‐bp fragment located upstream of the *CsTON2* coding region (*proCsTON2*) was amplified using PCR. Then the 35S promoter was replaced with *proCsTON2* using SalI and BstEII, and finally the *proCsTON2*:CsTON2‐Flag vector was generated. The obtained vectors were transformed into the *Agrobacterium tumefaciens* strain EHA105, and the cotyledon transformation method mediated by Agrobacterium was used for genetic transformation of cucumber (Hu et al. 2017). The primer information is listed in Dataset S4.

### 2.6 RNA Extraction and qRT‐PCR

RNA samples were extracted using the Eastep Super Total RNA Extraction Kit (Promega) and then the RNA was reverse transcribed into cDNA using the FastKing gDNA Removal RT Super Mix Kit (Tiangen, Beijing, China). RT‐qPCR analysis was performed using TB Green Premix Ex Taq II (Takara, Kyoto, Japan) on the CFX384 Real‐Time Fluorescence Quantitative PCR System (BIO‐RAD). The cucumber ubiquitin gene (*CsaV3_5G031430*) was used as the internal reference gene to calculate the relative expression of the detected genes (Wan et al. 2010). Each gene included three biological replicates and each biological replicate contained three technical replicates. The primer information is listed in Dataset S4.

### 2.7 In Situ Hybridization

Female flower buds of the cucumber inbred line XTMC were collected and fixed in formalin‐acetic acid‐alcohol (FAA) fixative solution, which was prepared as follows: 50 mL of anhydrous ethanol, 5 mL of glacial acetic acid, 10 mL of 37% formaldehyde and 35 mL of DEPC‐treated water (DEPC‐H_2_O). All operational steps and precautions were performed as described in the previously published method (Zhang et al. 2013; Gu et al. 2018). The sense and antisense probes were designed targeting the specific region of the *CsTON2* sequence and the primer sequences used for probe synthesis are listed in Dataset S4.

### 2.8 Cell Number and Cell Area Measurement

The samples from 10 DAA or 30 DAA cucumber fruits with a peel approximately 1 cm thick were subjected to paraffin embedding and fixation. Next, 10 μm thick transverse and longitudinal sections were prepared from the slices using a microtome. The sections were stained with toluidine blue and observed under a microscope (Olympus DP72, Japan). The cell number (X), cell area (A) and average cell area within a specified section were computed using ImageJ software. The area of the entire fruit transections and longitudinal sections (A') was found by measuring the fruit diameter and applying the formula for the area of a circle (in the case of transections) or an ellipse (for longitudinal sections). The cell number in the whole fruit transections and longitudinal sections (X') was derived using the formula X/A = X'/A' (Yang et al. 2013). For each fruit, three positions were chosen and six regions were selected for statistical analysis.

### 2.9 Split‐Luciferase Complementation Assay

The coding sequences of *CsSUN* and *CsTRM5* were respectively cloned and ligated into the pCAMBIA1300‐nLUC vector via homologous recombination. Meanwhile, *CsTON2*, *CsTON2* ^*m*^, *CsBSK1*, *CsBSK3*, *CsBSK5*, *CsMAPKK* and *CsMAPK* were inserted into the pCAMBIA1300‐cLUC vector using the same method. The constructs were transformed into *Agrobacterium tumefaciens* strain GV3101 and co‐infiltrated into *N. benthamiana* leaves. Then the plants were cultured under normal conditions for 2 days before signal detection. The luciferase substrate solution was sprayed onto the abaxial side of *N. benthamiana* leaves, which were then incubated in the dark for 5 min. Subsequently, imaging was performed using a living imaging system (Lumazone1300B, Roper) or a chemiluminescence instrument (MiniChemi 610, Sagecreation) with an exposure time of 5 min to capture the fluorescent signals. The relative luciferase activity was recorded in a 96‐well plate using a luminometer (Tecan Infinite F200). The primers used for vector construction are listed in Dataset S4.

### 2.10 Co‐Immunoprecipitation (IP) Assay

The full‐length CDSs of *CsTON2* and *CsBZR1* were inserted into the vectors pCAMBIA1300‐GFP respectively, resulting in the CsTON2‐GFP and CsBZR1‐GFP constructs. The coding sequence of *CsSUN* and *CsTRM5* was constructed into the pCAMBIA1300‐Flag. Subsequently, CsTON2‐GFP and CsBZR1‐GFP with CsSUN‐Flag (CsTRM5‐Flag) constructs were co‐transformed into *N. benthamiana* leaves by *Agrobacterium* (GV3101)‐mediated infiltration. After incubation for 48 h, plants were collected and processed for Co‐IP assay according to protocols described previously (Liu et al. 2023).

### 2.11 Yeast Two‐Hybrid Assay

The *CsTON2* gene was cloned into the pGBKT7 vector, while the coding sequences of *CsSUN* and *CsTRM5* were inserted into the pGADT7 vector. Subsequently, the bait and prey vectors were co‐transformed into the yeast two‐hybrid strain Y2H Gold, which was then cultured on yeast synthetic dropout medium lacking tryptophan and leucine (‐WL) for 2–3 days. After the yeast colonies grew normally, the yeast transformants were screened on an interaction‐selective medium lacking Trp, Leu and His (‐WLH) or lacking Trp, Leu, His and Ade (‐WLHA). The primer information is listed in Dataset S4.

### 2.12 Protoplast Transformation Assay

Cotyledons from 7‐day‐old cucumber seedlings were used. The lower epidermis was carefully removed and the exposed mesophyll tissue was incubated in an enzyme digestion buffer. The tissue was digested for 4–5 h at room temperature on a low‐speed shaker to release protoplasts. Isolated protoplasts (2 × 10^5^) were incubated with plasmid DNA (10–20 μg) in transformation buffer. PEG4000 was added to a final concentration of 20%–40% and the mixture was incubated for 15 min. The reaction was stopped by dilution with W5 solution, followed by centrifugation. Finally, the protoplasts were resuspended in W5 solution and cultured in the dark for 12–14 h for subsequent observation under a microscope.

### 2.13 Dephosphorylation Assay in Vitro and Phos‐Tag SDS‐PAGE

CsSUN‐Flag was transiently expressed in *N. benthamiana* leaves either individually or co‐expressed with CsTON2‐GFP. Subsequently, CsSUN‐Flag protein was enriched by immunoprecipitation with Flag beads and protein samples were analysed by 8% regular SDS‐PAGE with 40 μM Phos‐tag and 100 μM MnCl_2_. After electrophoresis for 3 h, the gels were washed using general transfer buffer added 10 mmol/L EDTA for 10 min with gentle agitation. Then the gels were washed using general transfer buffer for 10 min with gentle agitation. When the Mn^2+^ in the gels was washed out, the gels were used for immunoblotting analysis.

### 2.14 Construction of Near‐Isogenic Lines

*FS1.2* (a 161‐bp deletion in the first exon of the *CsSUN*), *FS2.1* (with a 1‐bp deletion in the *CsTRM5*) and *Cston2* (*sf5* mutant having the 6457 genetic background) were used as parents for crossing. The resulting F_1_ was continuously backcrossed with *Cston2* for four generations to produce the bc_4_ generation. During this process, background selection was conducted using molecular markers to ensure that the sites with different sequences remained heterozygous. Genotypic and phenotypic statistics were subsequently performed on the segregating population derived from the self‐crossing of the bc_4_ generation. The primer information of molecular markers is listed in Dataset S4.

### 2.15 Statistical Analysis

All experiments were replicated at least three times. Quantitative data were analysed using GraphPad Prism 10.1.2. Statistical analyses were performed using the software's built‐in tools and parameters: One‐Way ANOVA with Tukey's multiple comparisons test was used for pairwise comparisons among multiple samples and the *t*‐test was applied for comparisons between two samples.

### 2.16 Accession Number

Sequence data in this article can be found in Cucurbit Genomics Database (https://www.cucurbitgenomics.org/). Gene accession numbers are listed in Table S2.

## 3 Results

### 3.1 Phenotypic Characterisation of the *sf5* Mutant in Cucumber

**FIGURE 1:**
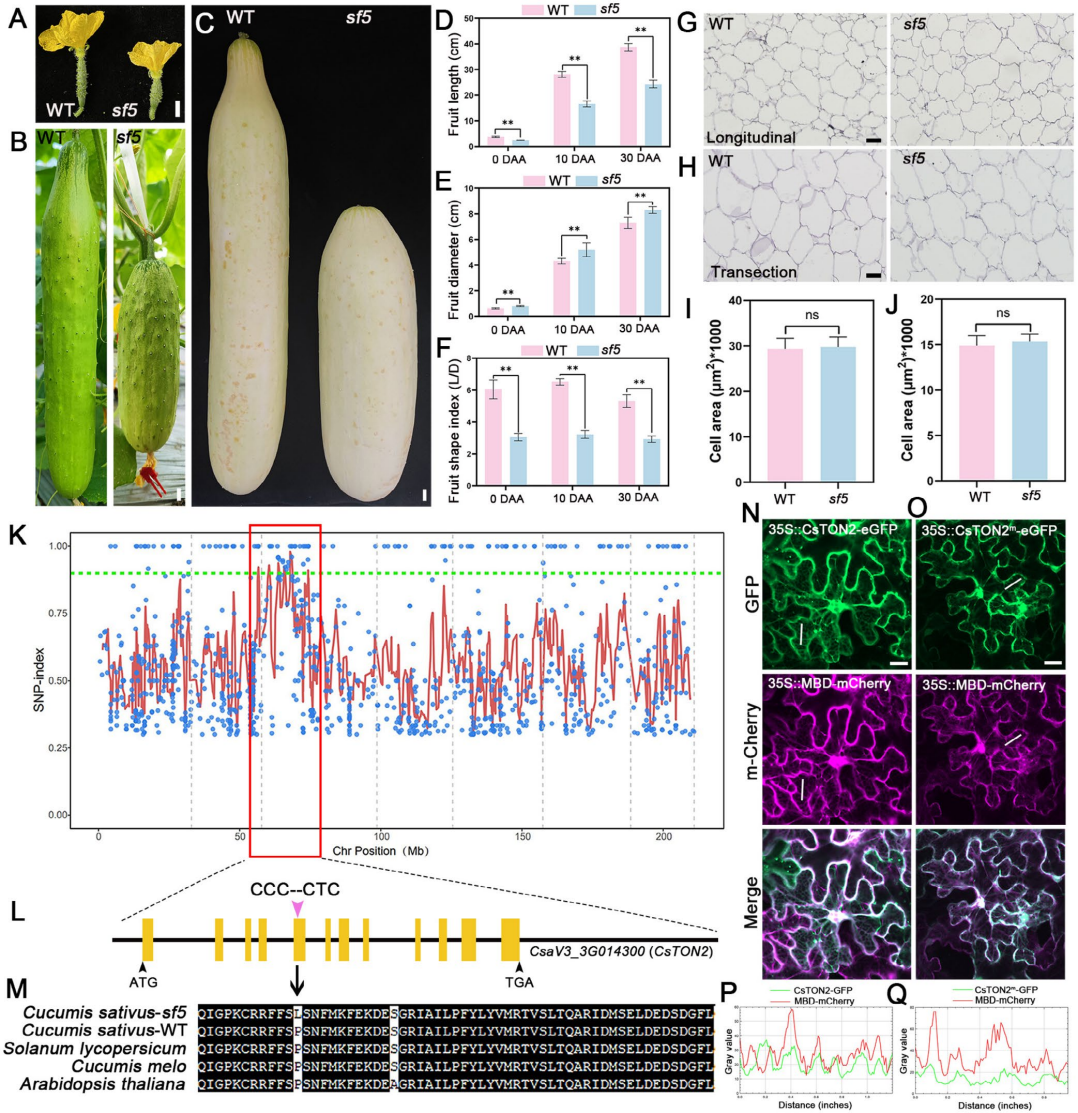
Phenotypic characteristics and gene cloning in *sf5* mutant. (A–C) Representative images of fruits from the wild‐type (WT) and the *sf5* mutant at anthesis (A), fruits at 10 days after anthesis (DAA) (B) and 30 DAA (C), scale bar = 1 cm. (D–F) Statistical data of fruit length (D), fruit diameter (E) and fruit shape index (F) at 0 DAA, 10 DAA and 30 DAA. Values are means ± SD, *n* ≥ 6. (G, H) Longitudinal (G) and transverse (H) pericarp sections of fruits at 30 DAA in WT and *sf5* mutants, scale bar = 100 μm. (I, J) Cell size statistical of the fruit pericarp in transverse and longitudinal sections of WT and *sf5* mutants. Values are means ± SD, *n* = three individual fruits. Significant differences are indicated by asterisks (*p < 0.05; and **p < 0.01, Student's *t*‐test). ns, no significant difference. (K) SNP index plot showing that the *sf5* locus is located on chromosome 3. (L) *CsaV3_3G014300* (*CsTON2*) gene structure. The yellow boxes represent exons. The SNP (C–T) change occurred in the fifth exon. (M) Alignment of *CsaV3_3G014300* homologues from different species. The arrow indicates the position of the mutated amino acid. (N, O) Subcellular localization of CsTON2 protein. The fusion proteins CsTON2‐GFP and CsTON2^m^‐GFP were co‐expressed with the microtubule marker MBD‐mCherry, respectively. Green fluorescence indicates the signals from GFP protein, red fluorescence indicates the signals from mCherry protein and white fluorescence shows the merged images with GFP and mCherry fluorescence. Scale bar = 20 μm. (P, Q) Colocalization analysis of CsTON2 or CsTON2^m^ with the microtubule marker MBD.

To identify more players during cucumber fruit shape specification, we isolated and characterised an EMS‐induced short fruit mutant, designated as *sf5* (short fruit 5). The *sf5* mutant exhibited significantly reduced fruit length and increased fruit diameter compared to the wild‐type 6457 line at various developmental stages (Figure 1A–C). Quantitative analyses revealed that *sf5* mutant showed 36.1%, 40.6% and 36.3% reduction in fruit length at 0, 10 days after anthesis (DAA) and 30 DAA, respectively. Conversely, fruit diameter increased by 29.0%, 20.0% and 13.7% at the corresponding stages. These morphological changes resulted in a significantly decreased FSI in *sf5* mutant (Figure 1D–F). To investigate the cause of fruit shape variation at the cellular level, we examined longitudinal and transection of the fruit pericarp at 30 DAA in both WT and *sf5* mutants. No significant difference in cell area was observed between WT and *sf5* mutant (Figure 1G–J). Moreover, seven cell division‐related marker genes were differentially expressed in WT and *sf5* fruits at anthesis (Figure S1A–H), suggesting that the altered fruit morphology in *sf5* may be due to cell division rather than cell expansion. Notably, the *sf5* mutant also exhibited shorter and wider cotyledons and seeds, similar to the fruit phenotype (Figure S2D–H). In addition, *sf5* mutant plant displayed reduced plant height, thicker stems and more round leaves compared to WT (Figure S2A–C).

### 3.2 Inheritance Analysis and Mapping of the Candidate Gene for *sf5* Mutant

Next, we performed genetic analysis using reciprocal crosses between the WT (long‐fruit) and *sf5* mutant (short‐fruit). All F₁ individuals from both reciprocal crossings (WT × *sf5* and *sf5* × WT) exhibited long‐fruit phenotype similar to that of WT, indicating complete dominance of the long‐fruit trait over the short‐fruit phenotype. In the F_2_ population of 253 individuals, 188 displayed long‐fruit while 65 showed short‐fruit. This segregation pattern fits the Mendelian ratio of 3:1 (χ^2^ = 3.841, Table S1), suggesting that the short‐fruit phenotype in *sf5* is controlled by a single recessive gene.

To identify the candidate gene responsible for the *sf5* phenotype, we performed whole‐genome resequencing and MutMap analysis. The analysis identified a single missense mutation with SNP‐index value of 1.0 located on chromosome 3 (Figure 1K) (DataSet S1). This mutation was mapped to the coding sequence of *CsaV3_3G014300*, encoding a B subunit of protein phosphatase 2A, which shows high sequence homology to Arabidopsis *TONNEAU2/FASS* (*TON2*). Sequence analysis revealed a C‐to‐T substitution at position 459 of the *CsaV3_3G014300* CDS sequences in *sf5* (Figure 1L), resulting in an amino acid change from proline (P) to leucine (L). This proline residue was evolutionarily conserved across plant species (Figure 1M). Therefore, *CsaV3_3G014300* was identified as the candidate gene of *sf5* and designated as *CsTON2* hereinafter. Phylogenetic analysis demonstrated that cucumber contains only a single *TON2* homologue (Figure S3A). The amino acid sequence of the TON2 protein is highly conserved across different species (DataSet S2).

Expression analysis showed that *CsTON2* transcripts were detected in fruits, lateral buds, male and female flower buds, shoot apex and leaves. Notably, *CsTON2* exhibited the highest expression in fruits compared to other tissues (Figure S3B). In situ hybridization further confirmed the enrichment of *CsTON2* transcripts in fruit ovules (Figure S3D–F). To investigate the subcellular localization of CsTON2, CsTON2‐GFP and CsTON2^m^ (the mutant version from *sf5*) ‐GFP fusion constructs were generated and co‐expressed with the Microtubule Binding Domain fused to mCherry (MBD‐mCherry) in *N. benthamiana* leaves. Our data revealed that the GFP signals from both CsTON2‐GFP and CsTON2^m^‐GFP overlapped with the red fluorescence of MBD‐mCherry (Figure 1N–Q). Additionally, we co‐expressed CsTON2‐GFP and CsTON2^m^‐GFP with CsSWEET7a‐mCherry (a previously reported cell membrane‐localized protein in cucumber) (Li et al. 2021) in *N. benthamiana* leaves that harboured a nuclear marker (nuclear‐mCherry). The data showed that both CsTON2 and CsTON2^m^ were localized to the nucleus and cell membrane (Figure S3G–H). These results suggested that mutation of *CsTON2* in *sf5* does not alter its subcellular localization. Furthermore, there was no difference in *CsTON2* expression level between WT and *sf5* mutant (Figure S3C).

### 3.3 CsTON2 Regulates Fruit Shape in Cucumber

**FIGURE 2:**
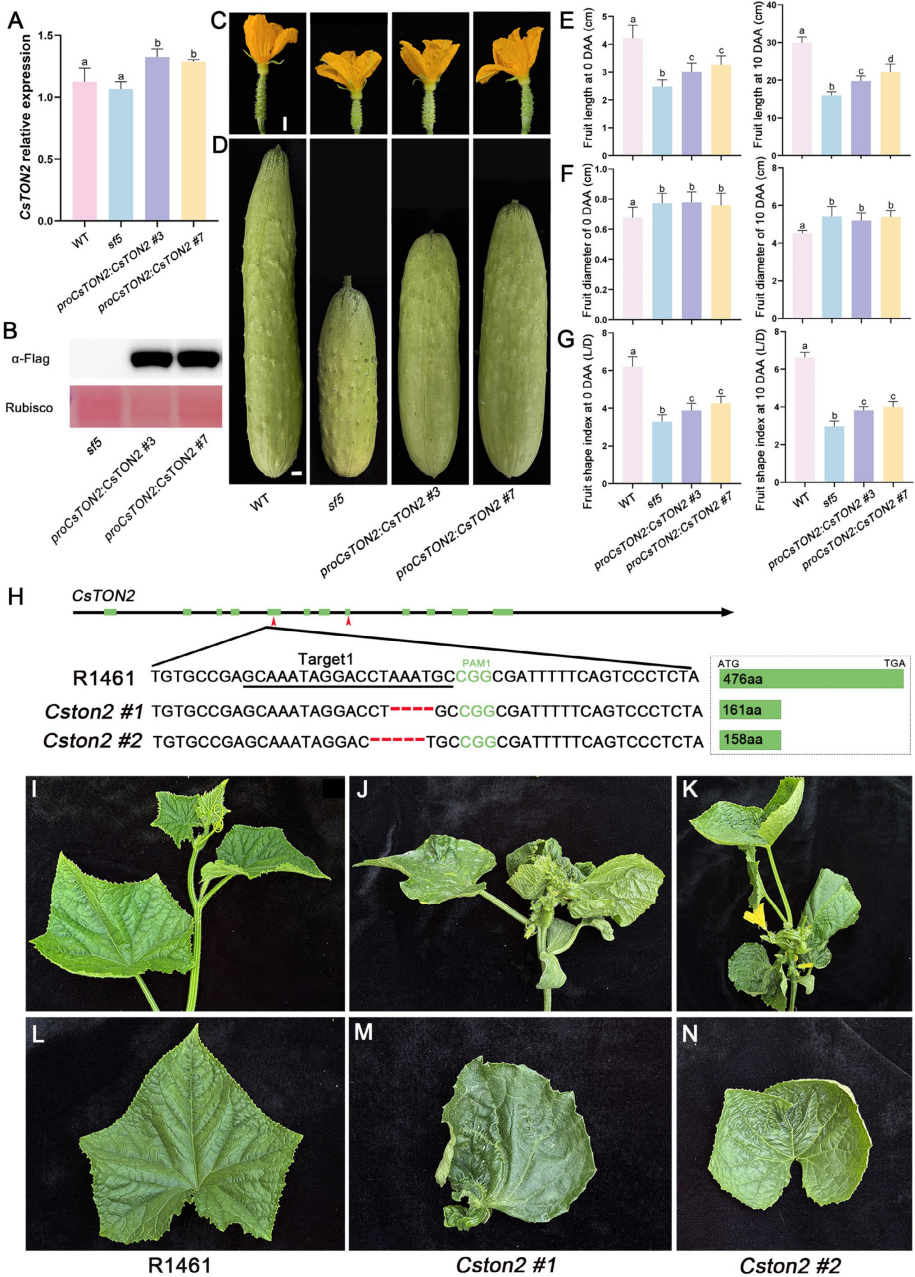
Functional characterisation of *CsTON2* in cucumber. (A, B) Expression and protein level analyses of *CsTON2* in complementary plants. CsUBI was used as internal standard. The CsTON2 protein was detected in lines using anti‐Flag antibody. The Ponceau S of Rubisco large subunit was demonstrated as a loading control. Data are the means ± SD of three independent biological replicates. (C, D) Representative images of fruits from WT, *sf5* and *proCsTON2*:CsTON2‐Flag plants at 0 DAA (C) and 10 DAA (D). Scale bar = 1 cm. (E–G) Quantification data showed the fruit length (E), fruit diameter (F) and the fruit shape index (G) of WT, *sf5* and *proCsTON2*:CsTON2‐Flag lines at 0 DAA and 10 DAA, respectively. Data are the means ± SD, *n* ≥ 6. Different letters represent significant differences by one‐way ANOVA with Tukey's post hoc test (*p* < 0.05). (H) Mutation forms of two homozygous *Cston2* mutants generated by CRISPR/Cas9 system. Both *Cston2 #1* and *Cston2 #2* were prematurely terminated. *Cston2 #1* encoded 161 amino acids and *Cston2 #2* encoded 158 amino acids. Target sequences and PAMs are marked. Deletion nucleotides are marked as a minus above the sequence. The dashed box shows the corresponding protein premature termination. The green boxes on the right indicate CsTON2 protein coding region in WT (R1461), *Cston2 #1* and *Cston2 #2*. (I–K) Local phenotypic of WT and *Cston2* mutant lines including the shoot apical meristem regions. (L–N) Leaf morphologies of WT and *Cston2* mutant plants.

To verify that *CsTON2* is the causative gene for the *sf5* phenotype, we introduced the full coding sequences (CDS) of *CsTON2* driven by its native 2‐kb promoter into the *sf5* mutant via *Agrobacterium*‐mediated transformation. Two independent transgenic lines (*proCsTON2*:CsTON2 #3 and *proCsTON2*:CsTON2 #7) were generated. Quantitative RT‐PCR analysis revealed around 1.3‐fold upregulation of *CsTON2* expression in transgenic lines compared to the *sf5* mutant (Figure 2A). Immunoblot assay confirmed the accumulation of CsTON2 protein in both transgenic lines (Figure 2B). Phenotypic characterisation showed that both complementation lines exhibited significantly increased fruit length at 0 and 10 DAA compared to the *sf5* mutant (Figure 2C,D). Quantitative analyses revealed that complementation lines showed a 23.9% and 39.1% increase in fruit length at 10 DAA compared to *sf5* mutant, while fruit width remained unchanged, leading to enlarged FSI (Figure 2E–G). Histological analysis revealed no significant differences in cell size among WT, *sf5* and complementation lines (Figure S4A,B,D,F). However, complementation lines exhibited a 20.5% and 46.9% increase in longitudinal cell numbers compared to *sf5* (Figure S4C), giving rise to the elongated fruit length. In contrast, transection cell numbers remained unchanged, consistent with unaltered fruit width (Figure S4E). These results suggested that *CsTON2* promotes fruit elongation by stimulating cell division. Consistent with the fruit phenotype, significant increases were observed in the length of seeds and cotyledons, as well as the corresponding ratio of length to width (Figure S4G–K). However, neither complementation lines fully rescued the phenotype to that of WT, indicating only partial complementation of the *sf5* mutant. To investigate the underlying reason, we analysed the activity of *CsTON2* promoters with different lengths. Notably, the 3‐kb promoter exhibited significantly higher activity than the 2‐kb version (Figure S4L), suggesting that the 2‐kb promoter used in our complementation assay may be insufficient to fully drive the *CsTON2* expression, which might account for the partial phenotypic rescue.

### 3.4 *CsTON2* Loss of Function Led to Severe Developmental Defects in Cucumber

To further elucidate the biological function of *CsTON2* in cucumber, we generated knockout mutants of *CsTON2* in the R1461 background using the CRISPR/Cas9 technology. Two independent lines, *Cston2 #1* and *Cston2 #2*, were selected for detailed phenotypic analysis. The *Cston2 #1* allele contained a 4‐bp deletion, while the *Cston2 #2* allele harboured a 5‐bp deletion in the target region. Both mutations resulted in premature stop codons (Figure 2H). The *Cston2 #1* and *Cston2 #2* mutant plants displayed severe morphological abnormalities including dwarfism, curled stem, embedded shoot apex and misshaped leaves (Figure 2I–N). Additionally, the trichome stalks on flowers and leaves were significantly shorter in *Cston2* mutants compared to R1461 (Figure S5A–F). These results demonstrated that *CsTON2* is essential for normal growth and development in cucumber.

### 3.5 CsTON2 Physically Interacts With CsTRM5 and CsSUN

**FIGURE 3:**
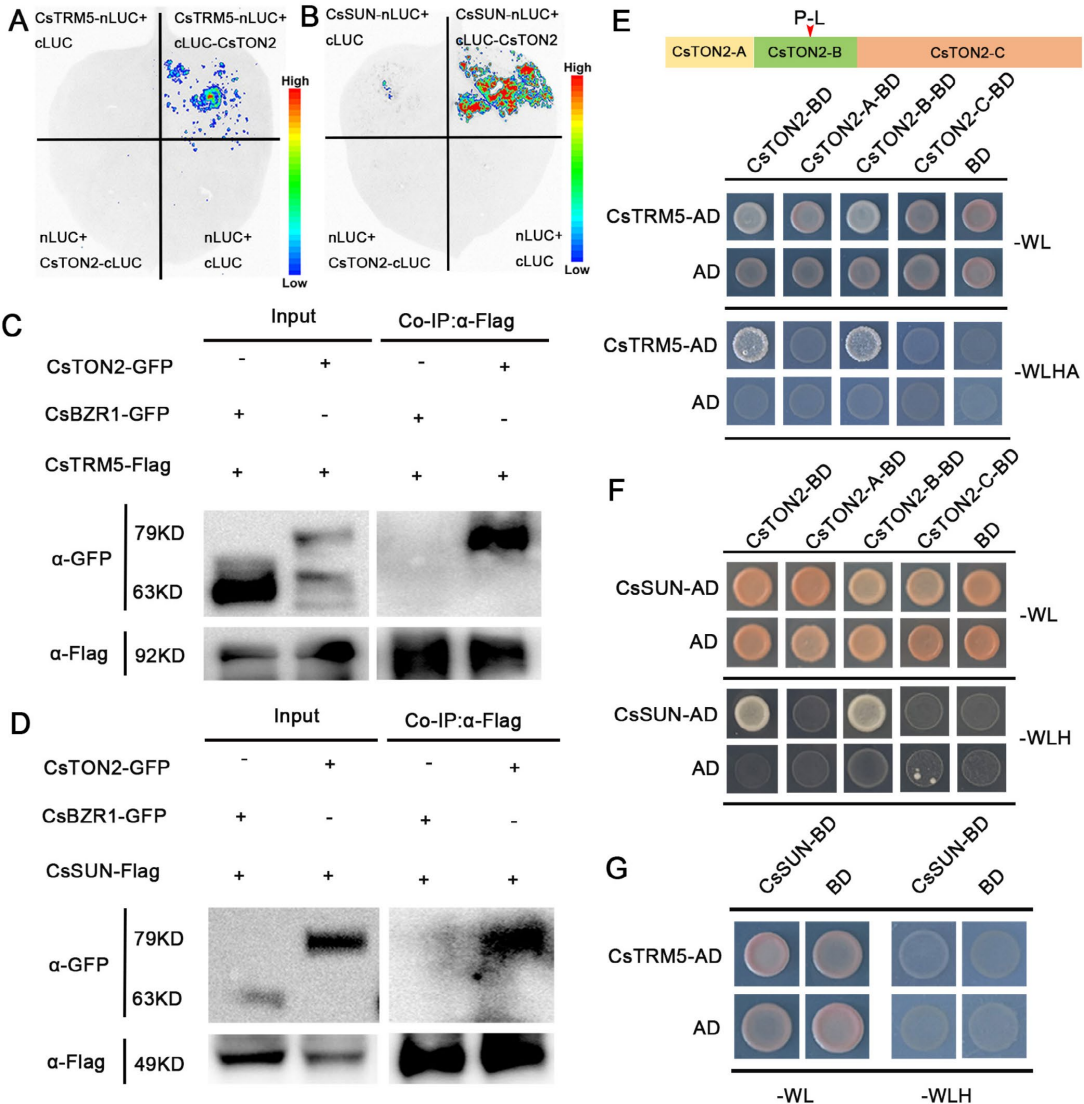
CsTON2 interacts with CsTRM5 and CsSUN at the protein level. (A, B) The interaction of CsTON2 with CsTRM5 (A) and CsSUN (B) revealed by firefly luciferase complementation imaging assay in *N. benthamiana* leaves. The luciferase proteins were fused to the N‐terminus of CsTRM5 or CsSUN and the C‐terminus of CsTON2, respectively. (C, D) The CsTON2‐CsTRM5 and CsTON2‐CsSUN interaction analysed by co‐IP assay in *N. benthamiana* leaves. CsTRM5 and CsSUN were immunoprecipitated by the anti‐Flag antibody. The eluted proteins were analysed using anti‐Flag or anti‐GFP antibodies. The Brassinazole‐Resistant 1 (CsBZR1)‐GFP was used as a negative control. (E–G) Yeast two‐hybrid assay revealing that CsTON2 interacts with CsTRM5 (E) and CsSUN (F), but no interaction between CsSUN and CsTRM5 (G). ‐WL indicates the medium lacking Leu and Trp. ‐WLHA indicates the medium lacking Leu, Trp, His and Ade. ‐WLH indicates the medium lacking Leu, Trp and His. The empty vectors (AD and BD) were used as negative controls.

Previous studies showed that TON2, a regulatory subunit of protein phosphatase 2A, directly interacts with TON1 and TRM family proteins to form the TTP complex that is required for the organization of microtubule arrays and PPB formation, and thus affects cell division pattern and cell growth (Camilleri et al. 2002; Drevensek et al. 2012; Spinner et al. 2013; Schaefer et al. 2017). CsTRM5 and CsSUN have been previously shown to regulate fruit shape by mediating cell division in cucumber (Pan et al. 2017; Xie et al. 2023). To investigate the relationships among CsTON2, CsTRM5 and CsSUN, we first performed luciferase complementation imaging (LCI) and coimmunoprecipitation (co‐IP) assays, which demonstrated that CsTON2 interacts with both CsTRM5 and CsSUN (Figure 3A–D). To further identify the interaction domains of CsTON2 with CsTRM5 and CsSUN, we generated a set of truncated CsTON2 fragments: CsTON2‐A (1–102 aa), CsTON2‐B (103–217 aa, containing the mutation site in *sf5*), CsTON2‐C (218–471 aa) and tested their interaction through yeast two‐hybrid (Y2H) assays. As expected, the CsTON2‐B fragment is required for CsTON2 to interact with CsTRM5 and CsSUN at the protein level (Figure 3E,F). However, no interaction was found between CsSUN and CsTRM5 (Figure 3G).

**FIGURE 4:**
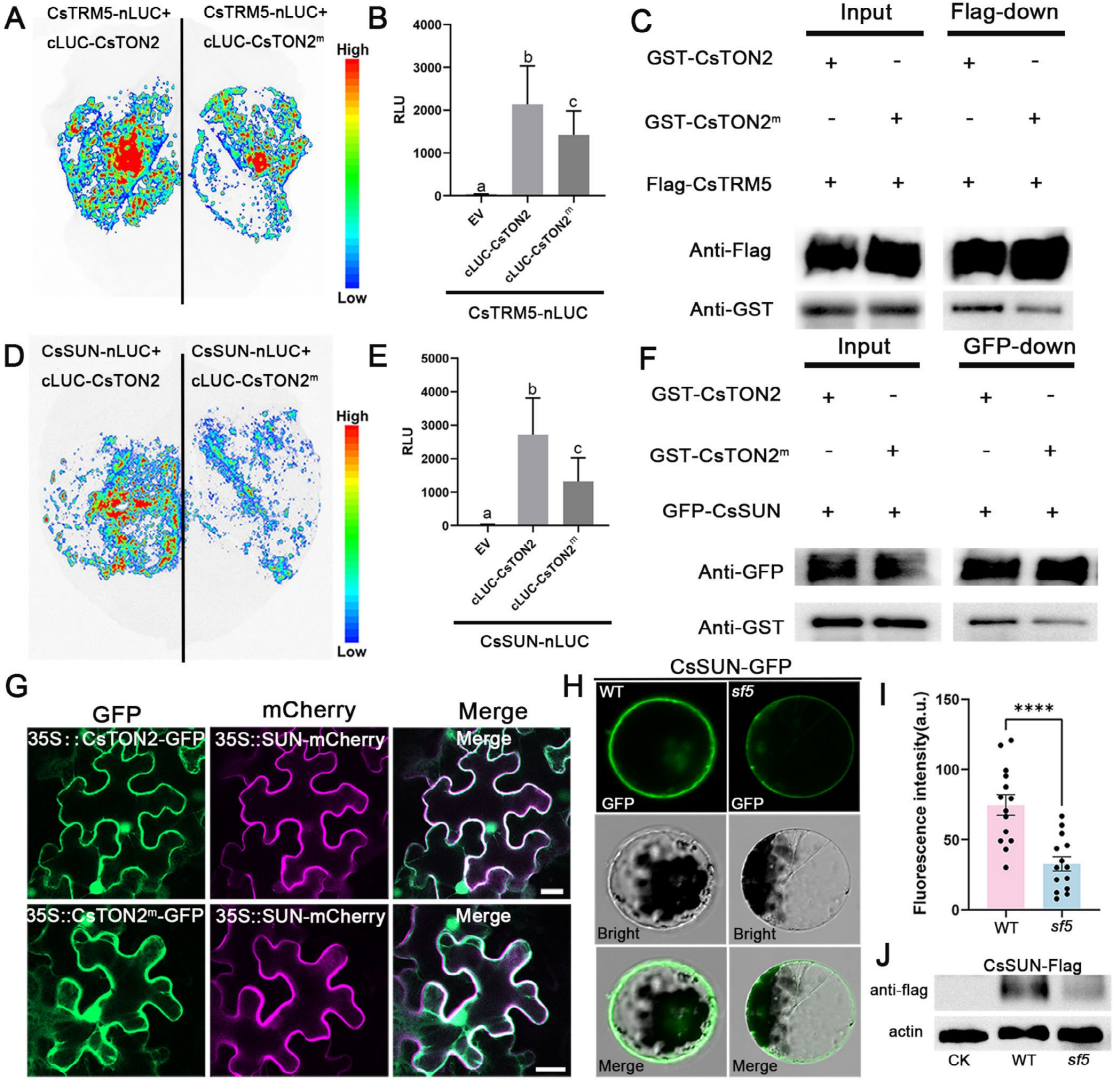
The CsTON2^m^ impairs the interaction with CsTRM5 and CsSUN. (A–F) Reduced interaction of CsTON2^m^ with CsTRM5 and CsSUN. The firefly luciferase complementation imaging assays were detected by a CCD camera (A, D) and the relative LUC activity was recorded by a luminometer (B, E). RLU, relative luminescence unit. Data are the means ± SD, *n* = 8. Different letters represent significant differences by one‐way ANOVA with Tukey's post hoc test (*p* < 0.05). Co‐IP assay between CsTON2 or CsTON2^m^ with CsTRM5 and CsSUN (C, F). The CsTRM5‐Flag and CsSUN‐GFP were expressed in *N. benthamiana* leaves. CsTON2‐GST and CsTON2^m^‐GST were obtained through in vitro induction. Anti‐Flag, anti‐GFP and anti‐GST antibodies were used to detect the corresponding fusion proteins. (G) Co‐expression of CsTON2 and CsTON2^m^ with CsSUN in *N. benthamiana* leaves epidermal cells. Green fluorescence indicates the signals from GFP protein, red fluorescence indicates the signals from mCherry protein and white fluorescence shows the merged images with GFP and mCherry fluorescence. Scale bar = 20 μm. (H) Expression of CsSUN‐GFP in WT and *sf5* cotyledon protoplasts. Scale bar = 20 μm. (I) Statistical analysis of the fluorescence intensity of CsSUN‐GFP in cotyledon protoplasts of WT and *sf5*. Values are means ± SD, *n* = 14. Asterisks indicate statistically significant differences (Student's *t*‐test, *p < 0.05, **p < 0.01, ***p < 0.001, ****p < 0.0001). (J) CsSUN‐Flag was expressed in cotyledons of WT and *sf5* and its protein expression was detected using anti‐Flag antibody. Actin was detected to indicate equal loading.

To explore why the SNP change in *sf5* mutant gave rise to the fruit shape phenotype, we compared the interaction intensity between CsTON2/CsTON2^m^ with CsTRM5 and CsSUN using LCI assays. The results showed that the chemiluminescence signals from the interactions between CsTON2^m^ and CsTRM5 or CsSUN were significantly weaker than that of CsTON2 (Figure 4A,D). Quantitative analysis showed that the interaction intensity of CsTON2^m^ with CsTRM5 and CsSUN decreased by 33.5% and 51.3%, respectively (Figure 4B,E). Moreover, semi‐in vitro pull‐down assays validated the reduced interaction of CsTON2^m^ with CsTRM5 and CsSUN (Figure 4C,F). Together, these results indicated that the SNP mutation in CsTON2 disrupts its interactions with CsTRM5 and CsSUN, thereby affecting fruit shape in cucumber.

### 3.6 CsTON2 Regulates the Protein Stability of CsSUN

As a regulatory subunit of PP2A, CsTON2 likely contributes to phosphatase activity by mediating substrate specification. To investigate whether CsSUN serves as a substrate of CsTON2, we first performed a co‐localization analysis. Our data showed colocalization of CsTON2 and CsSUN (Figure 4G). To further clarify their subcellular localization, we co‐expressed CsTON2 or CsSUN with cell membrane marker CsSWEET7a. The results revealed that both CsTON2 and CsSUN co‐localize to the cell membrane (Figure S6A,B). Next, we conducted a protoplast transformation assay to compare CsSUN protein level between WT and *sf5* mutant. Confocal imaging combined with quantitative analysis showed that the fluorescence intensity of CsSUN‐GFP was significantly higher in WT protoplasts than those of *sf5* (Figure 4H,I), implying that CsTON2 may regulate the stability of the CsSUN protein. To validate this notion, we transiently expressed CsSUN‐Flag in the cotyledons of WT and *sf5*. The results showed that the abundance of CsSUN protein in the *sf5* background was significantly lower than those in WT (Figure 4J). To investigate whether the reduced CsSUN abundance is caused by altered phosphorylation levels, we transiently expressed CsSUN alone or co‐expressed CsSUN with CsTON2 in *N. benthamiana* leaves. Our results showed that the level of phosphorylated CsSUN protein was significantly decreased in the presence of CsTON2 (Figure S7A), suggesting that CsTON2 may mediate the dephosphorylation of CsSUN. IP‐MS and LCI assays showed that CsSUN interacts with three BRASSINOSTEROID‐SIGNALLING KINASEs (BSKs) and two mitogen‐activated protein kinases (Figure S7B–F) (DataSet S3), implying that CsSUN may undergo dynamic phosphorylation and dephosphorylation. Taken together, these data indicated that the CsTON2 mutation in *sf5* impairs the interaction intensity with CsSUN, thereby reducing the protein stability of CsSUN.

### 3.7 CsSUN Positively Regulates Fruit Length in Cucumber

**FIGURE 5:**
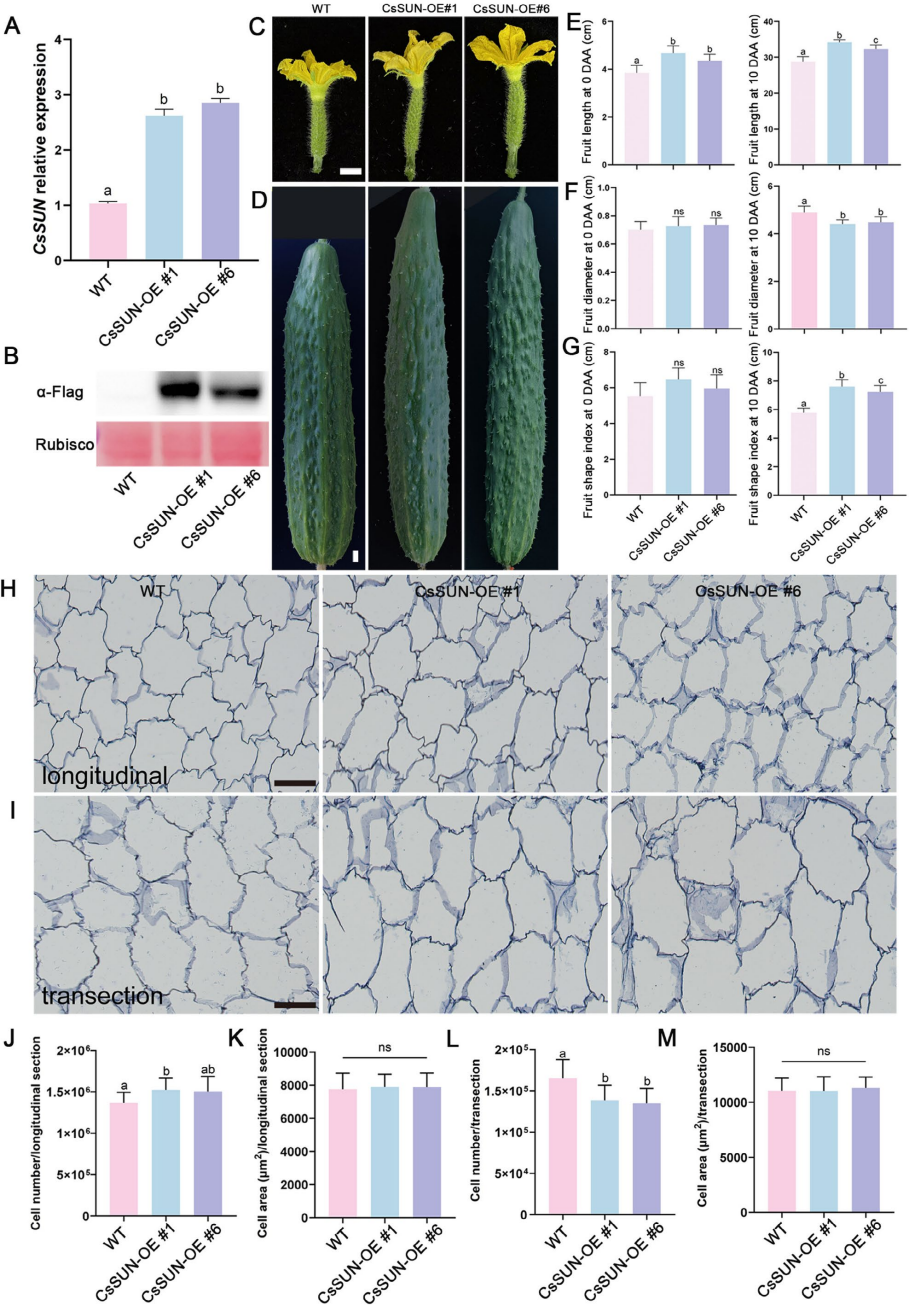
Phenotypic analysis of *CsSUN* overexpression lines. (A, B) Expression and protein level analysis of *CsSUN* in WT and CsSUN‐OE plants. CsUBI was used as internal standard. Data are the means ± SD of three independent biological replicates. Different letters represent significant differences by one‐way ANOVA with Tukey's post hoc test (*p* < 0.05). The CsSUN protein was detected in lines using anti‐Flag antibody. The Ponceau S of Rubisco large subunit was demonstrated as a loading control. (C, D) Representative images of fruits from WT and *CsSUN* overexpression plants at 0 DAA (C) and 10 DAA (D). Scale bar = 1 cm. (E–G) Quantification data showed the fruit length (E), fruit diameter (F) and the fruit shape index (G) of WT and *CsSUN* overexpression plants at 0 DAA and 10 DAA, respectively. Data are the means ± SD, *n* ≥ 6. Different letters represent significant differences by one‐way ANOVA with Tukey's post hoc test (*p* < 0.05). (H, I) Longitudinal sections (H) and transections (I) in the mesocarp at 10 DAA of WT and *CsSUN* overexpression plants. Scale bar = 100 μm. (J–M) Cell number and cell size statistics of the fruit pericarp in longitudinal sections (J–K) and transections (L–M) of WT and *CsSUN* overexpression plants. Data are the means ± SD, *n* ≥ 18. Different letters represent significant differences by one‐way ANOVA with Tukey's post hoc test (*p* < 0.05). ns, no significant difference.

To validate the function of CsSUN in regulating fruit shape, we overexpressed CsSUN in cucumber and obtained two independent transgenic lines (CsSUN‐OE #1 and CsSUN‐OE #6). qRT‐PCR analysis confirmed that *CsSUN* expression was significantly upregulated by 2.6‐ and 2.9‐fold in the overexpression (OE) lines, respectively (Figure 5A). Immunoblot assay further verified the accumulation of CsSUN protein in the OE lines (Figure 5B). Phenotypic characterisation showed that the fruits of CsSUN‐OE lines were significantly longer than those of WT at both 0 DAA and 10 DAA (Figure 5C,D). Quantification data revealed that fruit length in CsSUN‐OE #1 and CsSUN‐OE #6 increased by 21.6% and 13.0% at 0 DAA and by 19.1% and 12.4% at 10 DAA, respectively, compared to WT (Figure 5E). Additionally, fruit width in the OE lines was reduced by 9.8% and 8.4% at 10 DAA (Figure 5F). Consequently, the fruit shape index was significantly increased at 10 DAA in OE lines (Figure 5G). Histological sectioning analyses demonstrated that there were no significant differences in the cell size between OE lines and WT (Figure 5H,I,K,M). However, the number of longitudinal cells was increased, while that of transection cells was reduced in OE lines (Figure 5J,L). These results indicated that *CsSUN* positively regulates cucumber fruit shape by promoting cell division along the longitudinal direction while inhibiting cell division along the transverse axes.

### 3.8 Genetic Interaction of *CsTON2* With *CsTRM5* and *CsSUN* During Fruit Shape Determination in Cucumber

**FIGURE 6:**
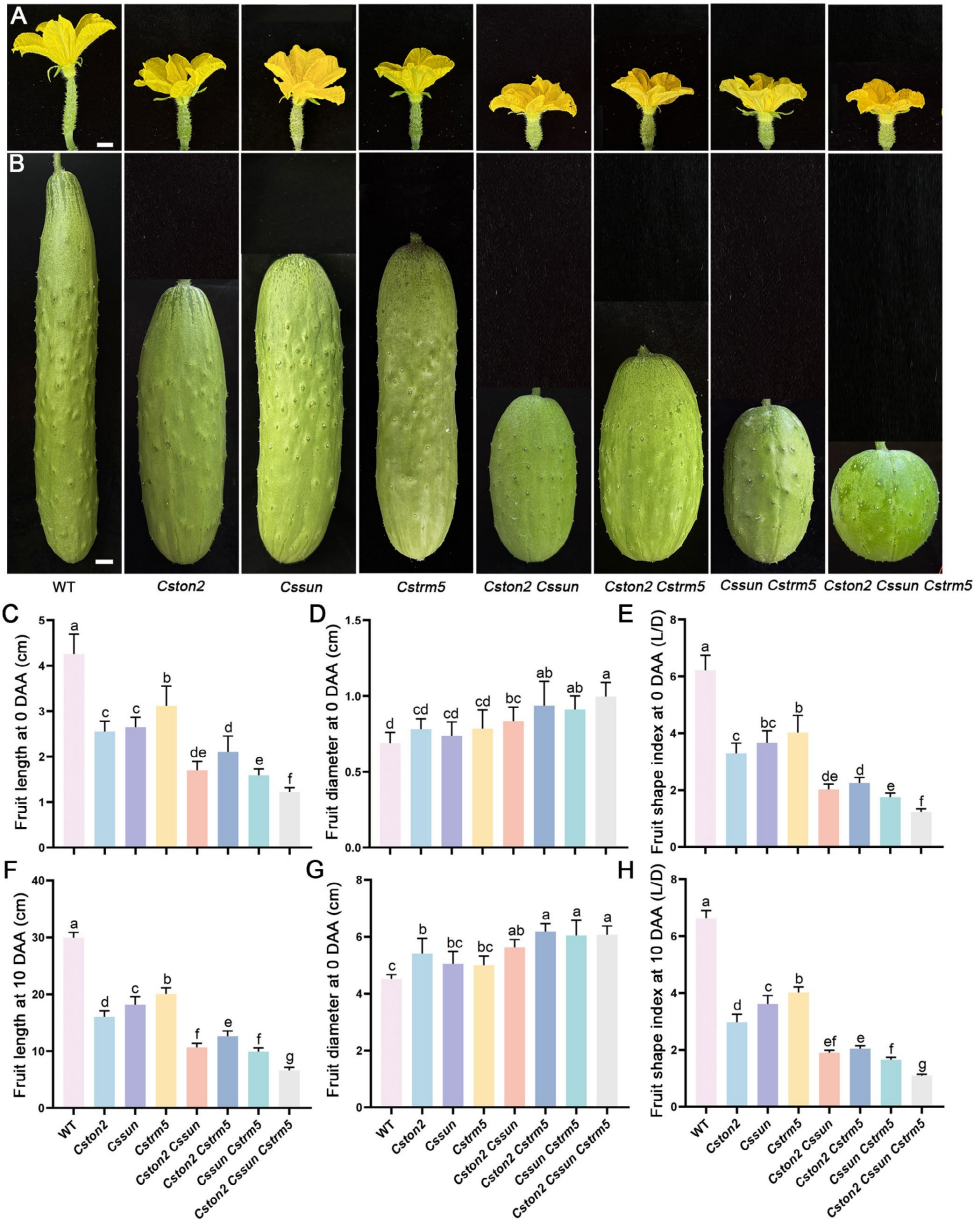
Genetic analyses of *CsTON2* with *CsTRM5* and *CsSUN* during fruit shape development in cucumber. (A, B) Representative images of fruits from WT, *Cston2*, *Cssun*, *Cstrm5*, *Cston2 Cssun*, *Cston2 Cstrm5*, *Cssun Cstrm5* and *Cston2 Cssun Cstrm5* mutant lines at 0 DAA (A) and 10 DAA (B). Scale bar = 1 cm. (C–H) Quantification data showed the fruit length (C, F), fruit diameter (D, G) and the fruit shape index (E, H) of the corresponding single, double and triple mutants at 0 DAA and 10 DAA, respectively. Data are the means ± SD, *n* ≥ 6. Different letters represent significant differences by one‐way ANOVA with Tukey's post hoc test (*p* < 0.05).

To explore the genetic relationship among *CsTON2, CsTRM5* and *CsSUN* during fruit development, we constructed near‐isogenic lines (NILs) by recurrent backcrossing the *FS1.2* (*Cssun* mutant) and *FS2.1* (*Cstrm5* mutant) into the *Cston2* (*sf5*) background (Figure S8A–D). Thus, we generated the single, double and triple mutants: *Cston2, Cssun, Cstrm5, Cston2 Cssun, Cston2 Cstrm5, Cssun Cstrm5* and *Cston2 Cssun Cstrm5* in the same genetic background. Phenotypic analysis revealed a progressive reduction in fruit length from single to double to triple mutants. Consistent with previous reports, the fruit length of *Cssun* and *Cstrm5* single mutants was significantly reduced at both 0 DAA and 10 DAA. Moreover, the double mutant displayed a more severe short‐fruit phenotype than the single mutants. Notably, the triple mutant exhibits the most pronounced phenotype, giving rise to a round‐shaped fruit (Figure 6A,B). Quantitative analyses revealed that the single mutants *Cston2, Cssun* and *Cstrm5* showed 46.6%, 39.4% and 33.0% reduction in fruit length, respectively. The double mutants *Cston2 Cssun, Cston2 Cstrm5* and *Cssun Cstrm5* displayed 64.5%, 57.8%, and 66.9% decrease in fruit length, respectively. However, the triple mutant *Cston2 Cssun Cstrm5* exhibited a 77.8% reduction in fruit length at 10 DAA (Figure 6C–H). Together, these data suggested that CsTON2, CsTRM5 and CsSUN coordinately regulate fruit shape formation in cucumber, probably functioning as a CsTRM5‐CsTON2‐CsSUN complex.

Next, we examined the histologic structures of longitudinal and transverse sections of the fruit pericarp at 10 DAA in WT, *Cston2*, *Cssun* and *Cstrm5* single mutants (Figure S9A,B). No significant difference in cell size was observed between WT and the mutants. However, the *Cston2*, *Cssun* and *Cstrm5* single mutants all showed a reduced number of cells along the longitudinal direction while an increased number of cells along the transverse axis (Figure S9C–F). These results were consistent with the shortened fruit length and increased fruit width observed in the mutants. Therefore, CsTRM5‐CsTON2‐CsSUN regulates fruit shape by modulating cell division direction in cucumber.

## 4 Discussion

### 4.1 CsTON2 Is a New Player for Fruit Shape Specification in Cucumber

Microtubule dynamics are essential for cell morphogenesis and organ shape development (Struk and Dhonukshe 2013). TON2 acts as a microtubule‐associated protein that governs cell division patterns and ultimately shapes organ morphology by organizing the cortical microtubule array (Kirik et al. 2012). In Arabidopsis, *ton2* mutants exhibit extreme phenotypes with a highly compressed apical‐basal axis (Camilleri et al. 2002). Furthermore, TON2 directly interacts with OFPs to influence microtubule reorientation in cotyledon pavement cells, thereby regulating leaf morphology (Zhang, Wu, et al. 2020). The rice TRM protein GW7 interacts with OsTON2 to regulate the shape of the leaf blade (Lee et al. 2006; Wang et al. 2015; Wu et al. 2023). In this study, we isolated a novel short and wide fruit mutant, *sf5*, resulting from a single‐nucleotide substitution in *CsTON2*. Introduction of CsTON2 can partially rescue the *sf5* fruit phenotype (Figure 2). Further, we found that CsTON2 localizes to microtubules and regulates fruit shape by affecting cell division (Figure 1 and Figure S1A–H). Thus, C*sTON2* is a novel microtubule‐associated regulator that functions in fruit shape development in cucumber.

### 4.2 CsTON2, CsTRM5 and CsSUN Coordinately Regulate Fruit Shape Through Mediating Cell Division Direction in Cucumber

**FIGURE 7:**
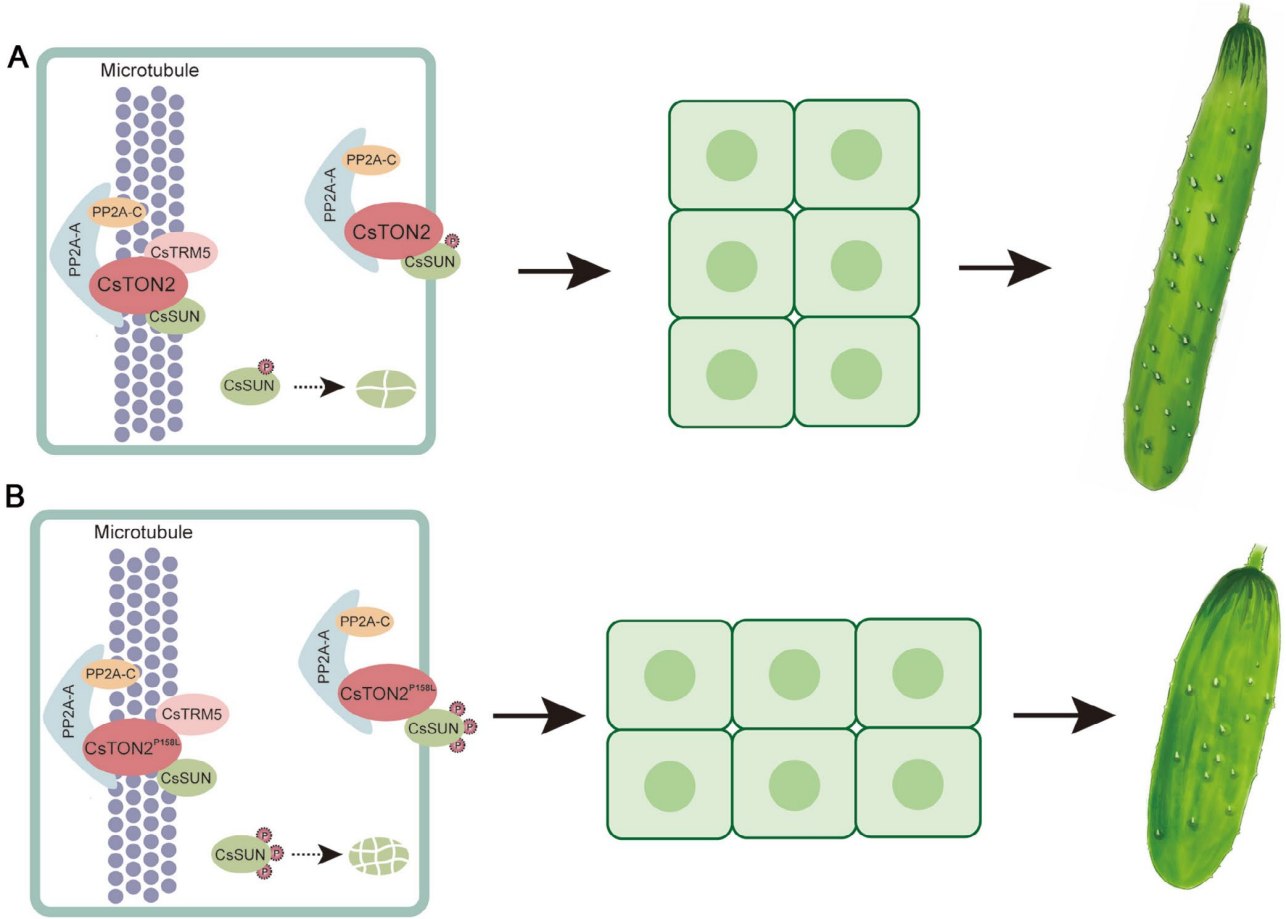
The working model of CsTRM5‐CsTON2‐CsSUN complex regulates fruit shape by mediating cell division direction in cucumber. (A) CsTON2 interacts with CsTRM5 and CsSUN. The interaction between CsTON2 and CsTRM5 facilitates the formation of a TTP complex in cucumber. CsTON2 directly interacts with CsSUN at the cell membrane, which may modulate the phosphorylation of CsSUN. (B) The SNP change in CsTON2 diminishes the interaction with CsTRM5 and CsSUN, potentially interfering with the assembly of the TTP complex, as well as leading to elevated phosphorylation of CsSUN and accelerated protein degradation. CsTON2 may act as a bridge between CsSUN and CsTRM5 to form a microtubule‐associated complex and to recruit CsSUN to microtubules, thereby regulating cucumber fruit shape by mediating the direction of cell division.

Previous studies have shown that TRMs protein play a critical role in organ shape regulation through assembly of the TTP complex in *Arabidopsis* and rice (Drevensek et al. 2012; Spinner et al. 2013; Wang et al. 2015). Here, we found CsTON2 interacts with CsTRM5 in cucumber (Figure 3), suggesting the interactions within TTP complex members are conserved. The SNP change in CsTON2 diminishes the interaction with CsTRM5, which may disturb TTP complex formation and affect cell division direction (Figure 7A,B). In tomato, SUN encodes an IQD family protein that localizes to microtubules and likely regulates fruit shape by promoting longitudinal cell division and inhibiting transverse cell division (Xiao et al. 2008; Wu et al. 2011; Bao et al. 2021). *CsSUN* has also been found to be the causal gene for *FS1.2*, a key locus for fruit shape variations in cucumber (Pan et al. 2017). Furthermore, we identified a direct interaction between CsTON2 and CsSUN at the cell membrane (Figure 4G). No interaction was found between CsSUN and CsTRM5 (Figure 3G), suggesting that CsTON2 may act as a bridge to interact with CsSUN and CsTRM5 to form a microtubule‐associated protein complex that controls fruit shape development in cucumber. Genetic analysis through single, double and triple mutants showed a progressive reduction in fruit length (Figure 6). Histologic analysis indicated that cell division, rather than cell expansion, was altered in these mutants, suggesting that CsTON2, CsTRM5 and CsSUN coordinately regulate fruit shape by mediating cell division direction in cucumber. However, due to technical difficulties, the specific cytological mechanisms through which the CsTRM5‐CsTON2‐CsSUN complex regulates microtubule assembly and organization to influence cell division and fruit shape await future investigation in cucumber. Further microtubule tracking observations (e.g., GFP‐TUA labeling) can be used to compare the dynamic changes of microtubule arrays between the wild type and transgenic lines.

The SNP change in CsTON2 diminishes the interaction with CsTRM5 and CsSUN (Figure 4A–F), and CsSUN protein abundance was significantly reduced in the *sf5* mutant (Figure 4H–J), implying decreased protein stability. As a regulatory subunit of PP2A, CsTON2 is likely to regulate the phosphorylation function of its substrates. In Arabidopsis, PP2A dephosphorylates and stabilizes SPEECHLESS to promote stomatal development (Bian et al. 2020). PP2A also interacts with KATANIN to regulate cuticular microtubule arrangement via dephosphorylation (Ren et al. 2022). In maize, the TRM homologue ZmLNG1 acts as a bridge between OFPs and the TTP complex to regulate organ morphology (leaves, tassels and ears) by influencing the phosphorylation level of OFPs (Wang et al. 2022). Here, our data showed that the interaction between CsTON2 and CsSUN decreased the phosphorylation level of CsSUN, thereby promoting its protein stability (Figure S7A). The impaired interaction in *sf5* may lead to elevated phosphorylation of CsSUN and increased protein degradation (Figure 7B). However, whether CsSUN stabilization is directly through dephosphorylation awaits further exploration. We also found that CsSUN interacts with BR signalling kinases CsBSKs and MAPK cascade kinases (Figure S7B–F). BSKs function not only as regulators of BR signalling but also as integrators of diverse endogenous and extracellular signals, thereby modulating multiple developmental processes (Zhao et al. 2025). MAPK‐mediated signal transduction plays a pivotal role in plant defence and developmental processes, such as resistance against brown planthoppers and grain size regulation (Guo et al. 2020; Wu et al. 2025). It would be interesting to identify the kinases responsible for phosphorylating CsSUN and elucidate how they cooperate with PP2A to regulate the phosphorylation‐dephosphorylation cycles of CsSUN. This regulatory balance may be essential for microtubule reorganisation and dynamic remodelling of microtubule arrays during cell division.

### 4.3 Utilisation of the SNP of *TON2* in Crop Breeding

Base editing, a precise genome editing technology, plays a pivotal role in improving agronomic traits in crops. This technique enables direct targeting of functional genes that regulate multiple traits through accurate base substitution, thereby specifically enhancing yield, quality and stress resistance (Li et al. 2024). Studies have demonstrated that targeted editing of *GmJAGGED1* from G to C in the EAR motif significantly increases seeds per plant and consequently improves single‐plant yield in soybean (Cai et al. 2021). In maize, *ZmLG1* encodes an SBP (SQUAMOSA PROMOTER‐BINDING)‐like transcription factor. Base editing of *ZmLG1* has successfully generated compact maize lines, providing novel germplasm for high‐density planting (Li et al. 2017).

In this study, we found that knockout of *CsTON2* resulted in severe developmental defects and dwarfism (Figure 2H–N), similar to that of the null mutant phenotype of *TON2* in Arabidopsis (Camilleri et al. 2002), which prevents its utilisation in crop breeding. Notably, we found a SNP change in *CsTON2* resulted in shorter and wider fruits with no obvious developmental abnormality. The SNP in *CsTON2* not only helps elucidate the regulatory mechanism of fruit development but also provides a practical molecular marker for breeding cucumber varieties with desired fruit shape. Cucumber fruit shape is a quantitative trait governed by a network of genes, including those harbouring natural SNPs, such as *CsFUL1*, *CsCRC* and *CsSEP2*, which play important roles in modulating fruit length (Zhao et al. 2024). The *CsFUL1* ^*A*^ allele is specifically present in East Asian cucumbers with long fruits that inhibit fruit elongation (Zhao et al. 2019). The *CsCRC* ^*G*^ allele promotes fruit length variations through stimulation cell expansion (Che et al. 2023). Knockout of *CsSEP2* resulted in severe floral developmental defects, while the SNP variations in the coding region of *SEP2* in both cucumber and melon specifically influence fruit length (Song et al. 2024). Therefore, the SNPs present in *CsTON2*, *CsFUL1*, *CsCRC* and *CsSEP2* can be utilised to modify fruit shape through CRISPR‐mediated base editing, so that to meet the diversified shape requirements from different ecotypes, production systems and consumer preferences. A typical application would be targeting the SNP site in *CsTON2* responsible for the *sf5* mutation: designing a single‐base editor to achieve precise base substitution, which enables rapid acquisition of the short and wide fruit phenotype.

In addition to the fruit shape alternation, the SNP change in *sf5* exhibited significant improvement in plant architecture, including reduced plant height, thicker stems, smaller leaves and a more compact growth habit; these architectural traits may enhance light use efficiency and facilitate mechanical harvesting, thus possess application potential in high‐density cucumber production.

## Author Contributions

J.Z., X.Z., and M.L. designed the research; M.L., X.L., Y.H. and C.L. performed the experiments; M.L., C.G. and C.S. analysed the data and participated in the experimental design; J.Z., X.Z. and M.L. wrote the paper; X.M., Y.G., S.H., L.H., L.L., Y.L., Z.L., D.S., Z.Z., X.S., Y.P., L.Y. and Y.H. provided experimental assistance; and all the authors read and approved the manuscript.

## Funding

This work was supported by the National Natural Science Foundation of China, 32025033, 32372699, 2115 Talent Development Program of China Agricultural University, 111 Project, B17043, Key Technology Research and Development Program of Shandong Province, 2024LZGCQY006 and Pinduoduo–China Agricultural University Research Fund (PC2023B01002).

## Conflicts of Interest

The authors declare no conflicts of interest.

## Supporting information


**Figure S1:** Expression analysis of genes involved in cell division.
**Figure S2:** Additional developmental phenotypes of WT and sf5.
**Figure S3:** Phylogenetic and expression analysis of CsTON2.
**Figure S4:** Histological and phenotypic analyses of CsTON2 complementation lines.
**Figure S5:** Mutation of CsTON2 resulted in abnormal trichome development in cucumber.
**Figure S6:** Subcellular localization of CsSUN and CsTON2.
**Figure S7:** CsTON2 regulated CsSUN phosphorylation and CsSUN‐interacting kinase proteins.
**Figure S8:** Construction of Cssun and Cstrm5 NILs.
**Figure S9:** CsTON2, CsTRM5 and CsSUN regulate fruit shape by influencing cell division.

**Table S1:** Inheritance analysis of fruit shape in sf5 mutant in cucumber.
**Table S2:** Genes information used in this study.

**DataSet: S1.** Candidate genes of *sf5*.
**DataSet: S2** Amino acid sequence of TON2 in different species.
**DataSet: S3** IP‐MS data of CsSUN.
**DataSet: S4** List of primers used in this study.

## Data Availability

The data that supports the findings of this study are available in the Supporting Information of this article.
