# Supplementary material for: The Microtubule‐Associated Protein CsTON2 Interacts With CsTRM5 and CsSUN to Regulate Fruit Shape Development in Cucumber
Source: Plant Biotechnol J. 2025 Dec 29;24(4):2725–41. doi: 10.1111/pbi.70519 (PMC13140649; doi:10.1111/pbi.70519)
Supplement: Supplementary file 1 — Figure S1: Expression analysis of genes involved in cell division. Figure S2: Additional developmental phenotypes of WT and sf5. Figure S3: Phylogenetic and expression analysis of CsTON2. Figure S4: Histological and phenotypic analyses of CsTON2 complementation lines. Figure S5: Mutation of CsTON2 resulted in abnormal trichome development in cucumber. Figure S6: Subcellular localization of CsSUN and CsTON2. Figure S7: CsTON2 regulated CsSUN phosphorylation and CsSUN‐interacting kinase proteins. Figure S8: Construction of Cssun and Cstrm5 NILs. Figure S9: CsTON2, CsTRM5 and CsSUN regulate fruit shape by influencing cell division. [file PBI-24-2725-s002.docx]

**
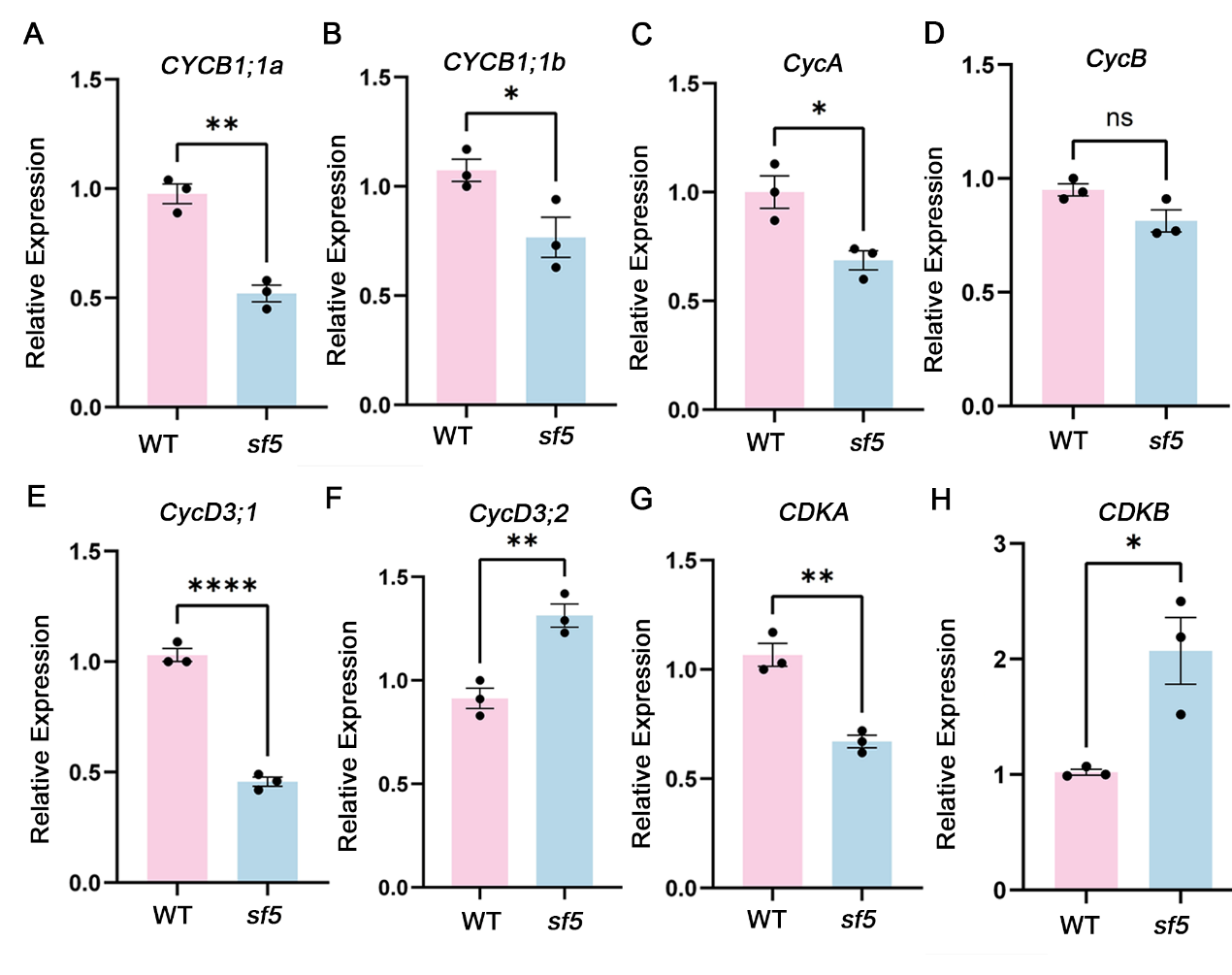
**

**Figure S1. Expression analysis of genes involved in cell division.**

(A-H) Expression analysis of *CYCB1;1a*, *CYCB1;1b*, *CycA*, *CycB*, *CycD3;1*, *CycD3;2*, *CDKA*, *CDKB*, in WT and *sf5*. Expression data were normalized using UBI. Data are the means ± SD of three independent biological replicates. A student’s *t*-test was performed, and statistically significant differences were indicated by *, *P* < 0.05; **, *P* < 0.01; ***, *P* < 0.001; ****, *P* < 0.0001; ns, no significant difference.

**
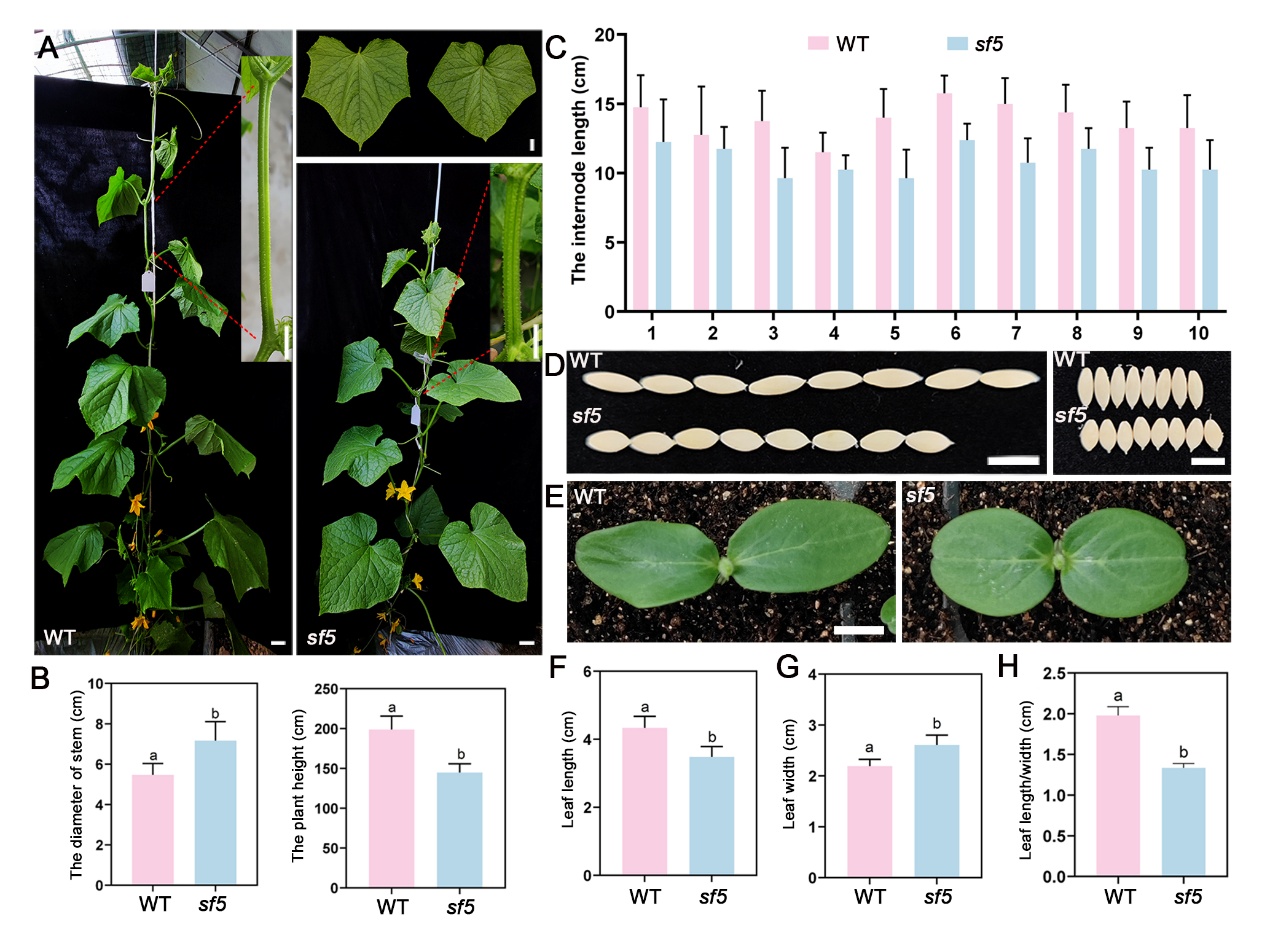
Figure S2. Additional developmental phenotypes of WT and *sf5*.**

(A) The phenotypes of plant height and stem diameter in the WT and *sf5*. Scale bar = 3.5 cm. (B-C) Statistics on stem diameter, plant height (B) and the internode length (C) from the 1^st^ to the 10^th^ node in WT and *sf5*. (D-E) Morphology of seeds (D) and cotyledons (E) in WT and *sf5* mutant lines. Scale bar = 1 cm. (F-H) Quantification of the length (F), width (G) and length / width (H) of cotyledons from WT and *sf5* mutant lines. Data are the means ± SD, n ≥6. Different letters represent significant differences by one-way ANOVA with Tukey’s post hoc test (*P* < 0.05).


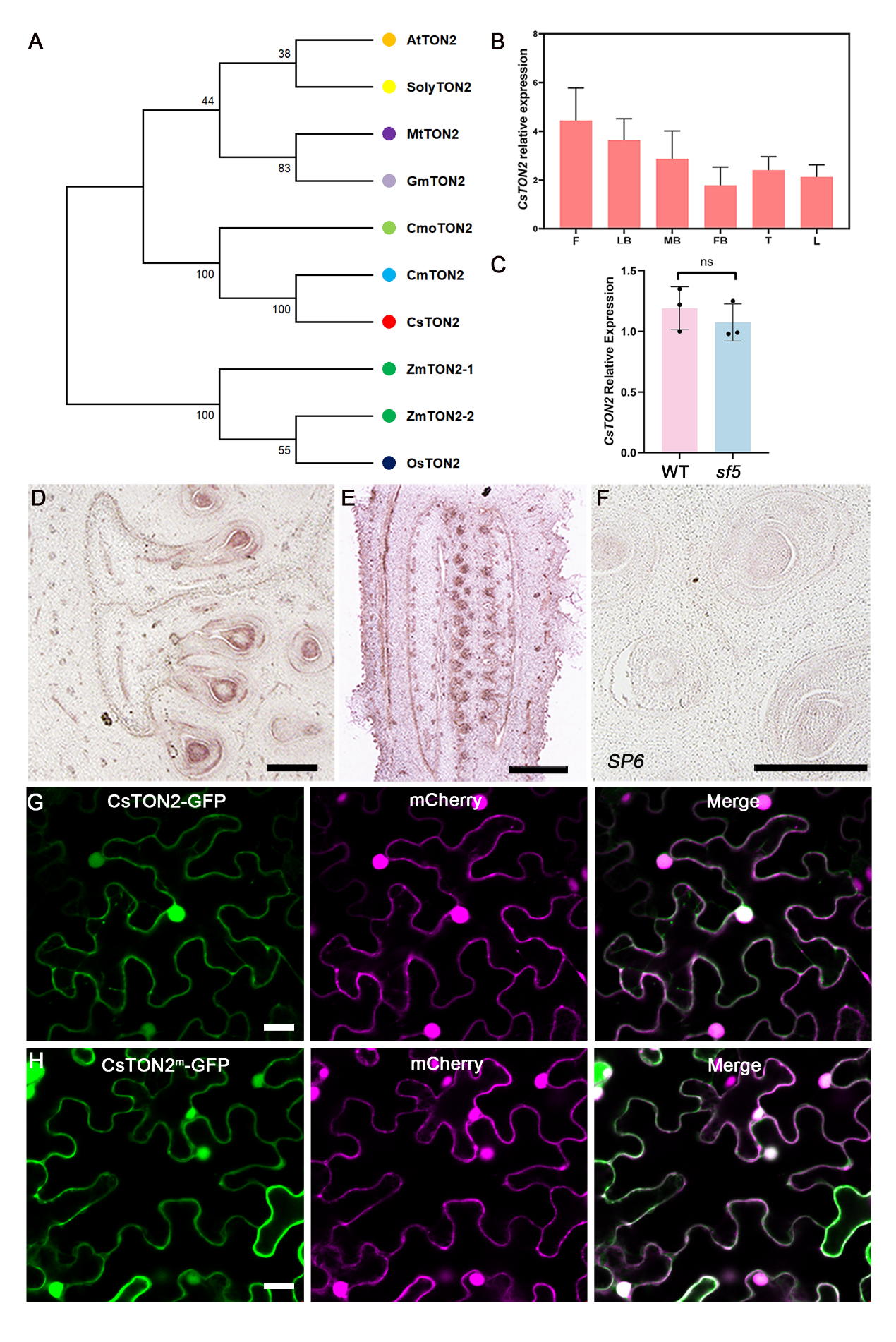


**Figure S3. Phylogenetic and expression analysis of *CsTON2*.**

(A) Phylogenetic tree analysis of CsTON2 by maximum likelihood method. (B) Expression analysis in different cucumber tissues by qRT-PCR*.* F: fruit, LB: lateral bud, MB: male bud, FB: female bud, T: shoot apex, L: leaves. CsUBI was used as internal standard. (C) Relative expression of *CsTON2* in WT and *sf5.* Data are the means ± SD of three independent biological replicates. Significance analysis was conducted with the two-tailed Student’s *t*-test (**P* < 0.05 and ***P* < 0.01). Values are means ± SD (n = 3). ns, no significant difference. (D-F) *In situ* hybridization analysis of *CsTON2*. The expression signals of *CsTON2* were enriched in the ovule (D) and female flower buds (E). The *CsTON2* sense probe SP6 was hybridized as a negative control (F). Scale bars = 200 μm. (G-H) Subcellular localization of CsTON2 protein. The fusion proteins CsTON2-GFP and CsTON2^m^-GFP were co-expressed with the cell membrane marker CsSWEET7a-mCherry in *N.benthamiana* leaves that harbored a nuclear marker (nuclear-mCherry), respectively. Green ﬂuorescence indicates the signals from GFP protein, red ﬂuorescence indicates the signals from mCherry protein and *N.benthamiana* leaves, and white ﬂuorescence shows the merged images with GFP and mCherry ﬂuorescence. Scale bar = 20 μm.


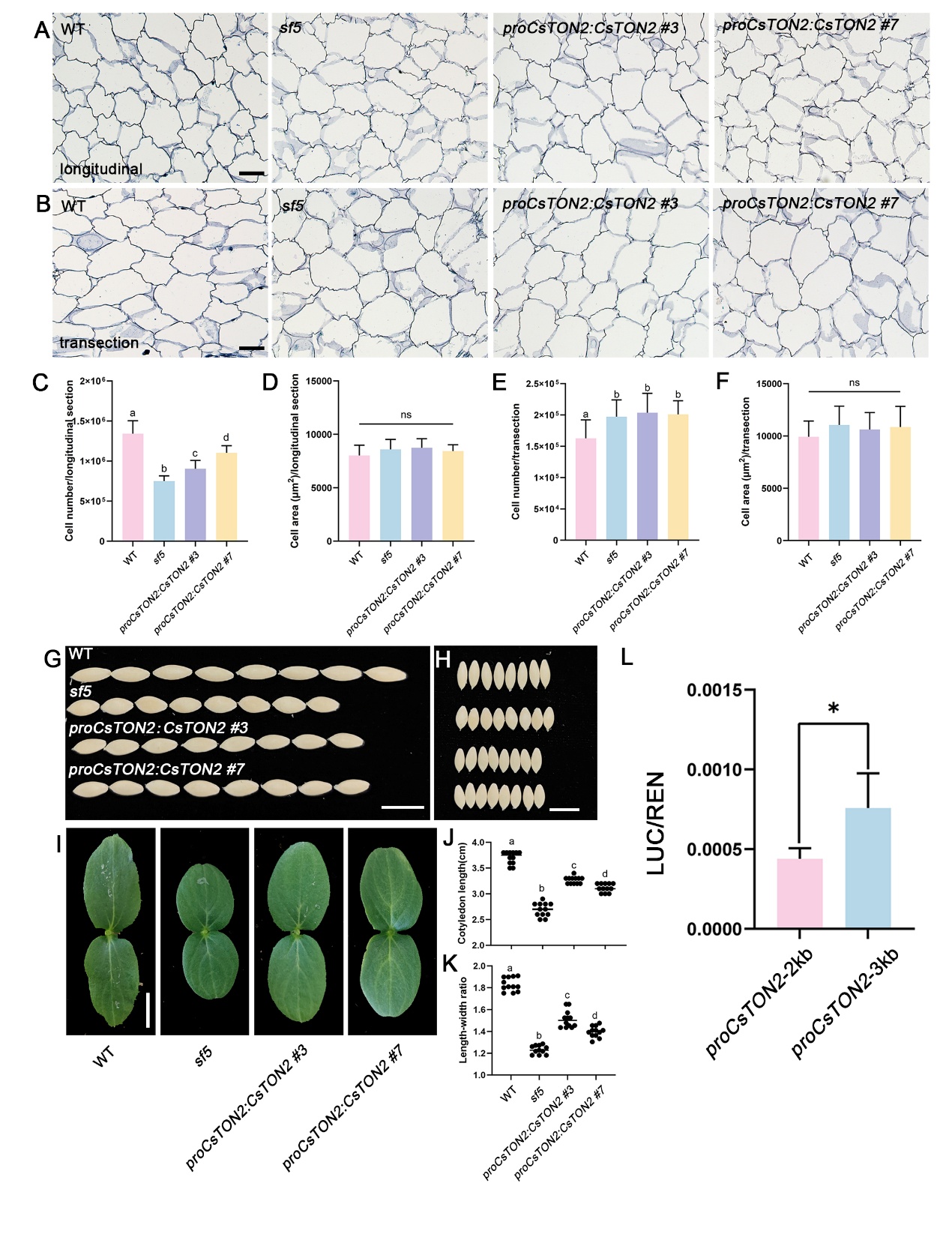


**Figure S4. Histological and phenotypic analyses of *CsTON2* complementation lines.** (A-B) Longitudinal (A) and transverse (B) sections in the mesocarp at 10 DAA of WT, *sf5* and *proCsTON2:*CsTON2-Flag lines. Scale bars = 100 μm. (C-F) Cell number and cell size statistical of the fruit pericarp in longitudinal (C-D) and transverse (E-F) sections of WT, *sf5* and *proCsTON2:*CsTON2-Flag lines. Data are the means ± SD, n ≥ 18. (G-I) Morphology of seeds (G-H) and cotyledons (I) in WT, *sf5* and *CsTON2* complemented lines. Scale bar = 1cm. (J-K) Quantification of the length (J) and length/width (K) of cotyledons. Data are the means ± SD, n = 12. Different letters represent significant differences by one-way ANOVA with Tukey’s post hoc test (*P* < 0.05). (L) Analysis of *CsTON2* promoter activity. Dual-LUC reporter analysis indicated that the 3kb *CsTON2* promoter had stronger activity than the 2 kb promoter. Significance analysis was conducted with the two-tailed Student’s *t*-test (**P* < 0.05 and ***P* < 0.01). Values are means ± SD (n = 9).


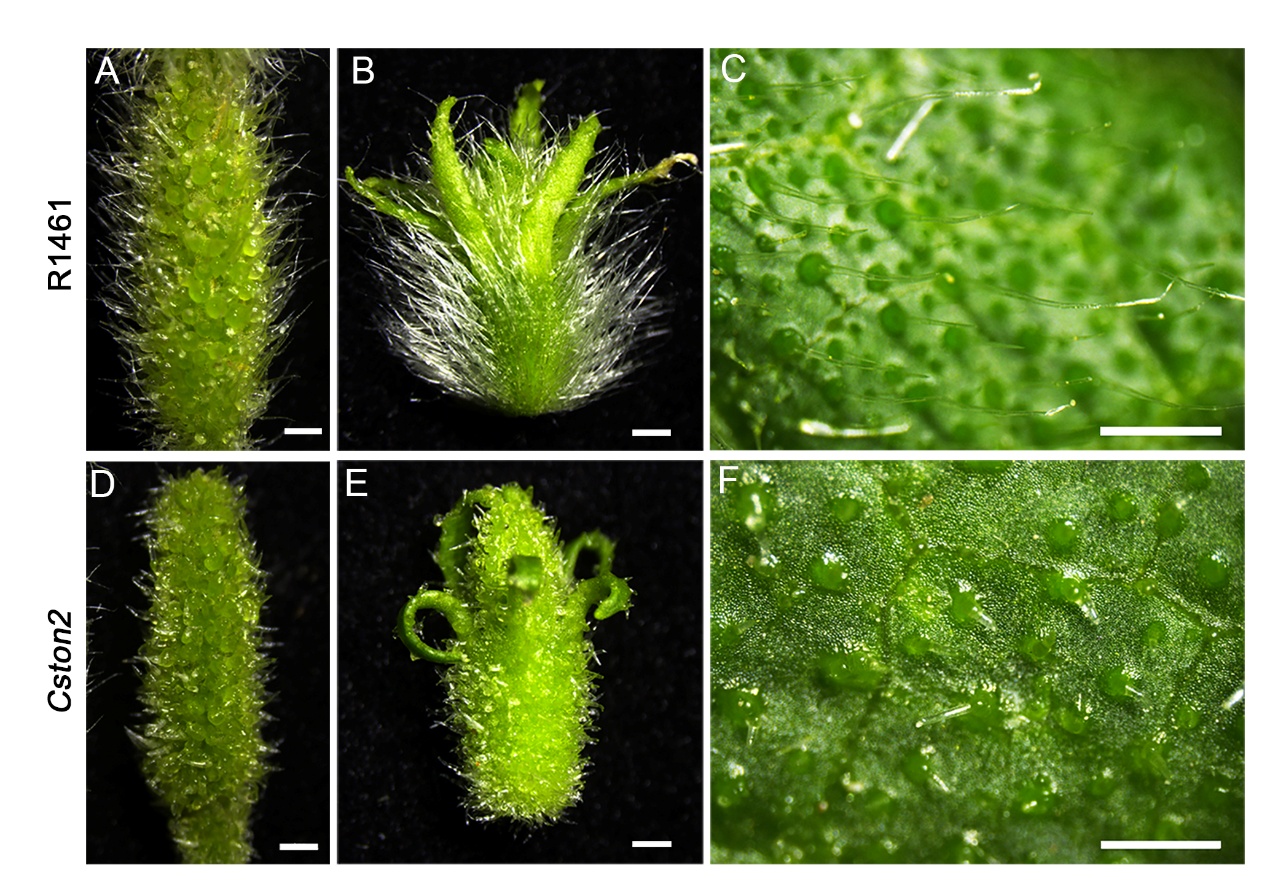


**Figure S5. Mutation of *CsTON2* resulted in abnormal trichome development in cucumber.**

(A-F) Trichomes on female flower buds, male flower buds, and leaves of WT (R1461) (A-C) and *Cston2* mutant (D-F). Scale bars =1 mm.

**
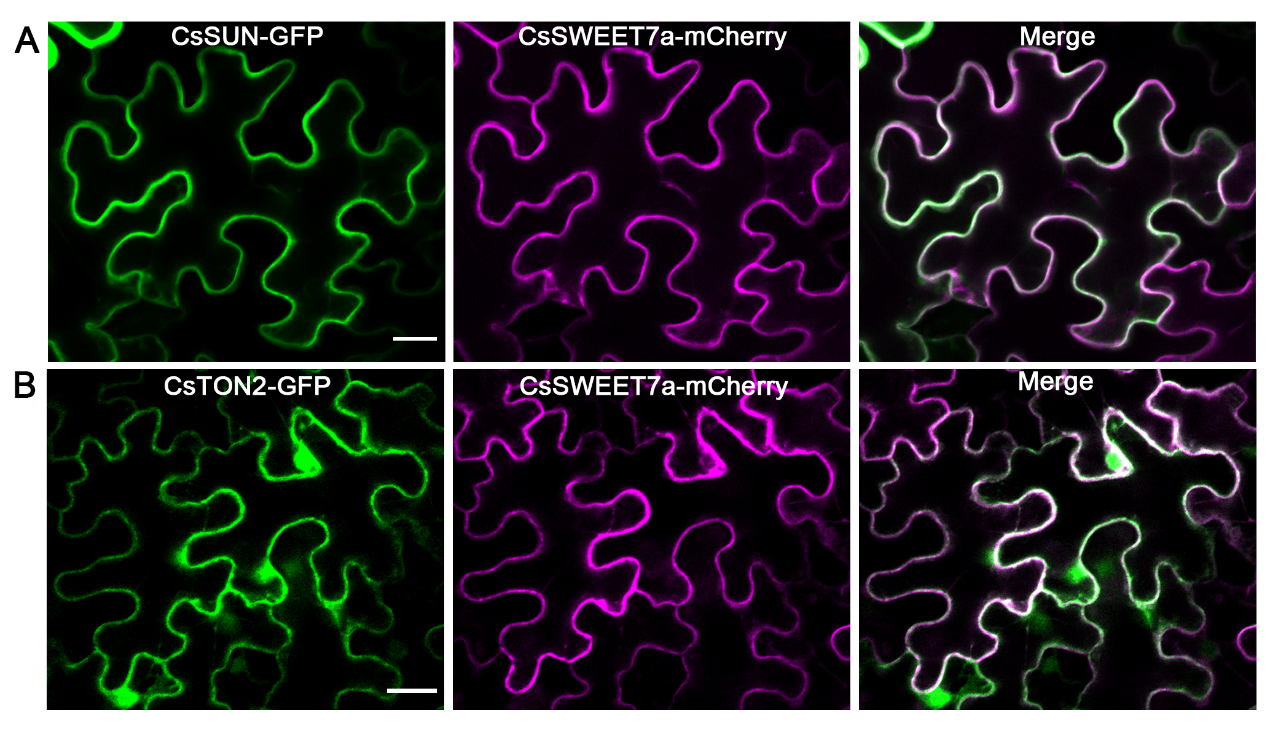
**

**Figure S6. Subcellular localization of CsSUN and CsTON2.**

(A-B) CsSUN-GFP and CsTON2-GFP were expressed in *N.benthamiana* leaves with CsSWEET7a-mCherry, respectively. Green ﬂuorescence indicates the signals from GFP protein, red ﬂuorescence indicates the signals from mCherry protein, and white ﬂuorescence shows the merged images with GFP and mCherry ﬂuorescence. Scale bars =20 μm.


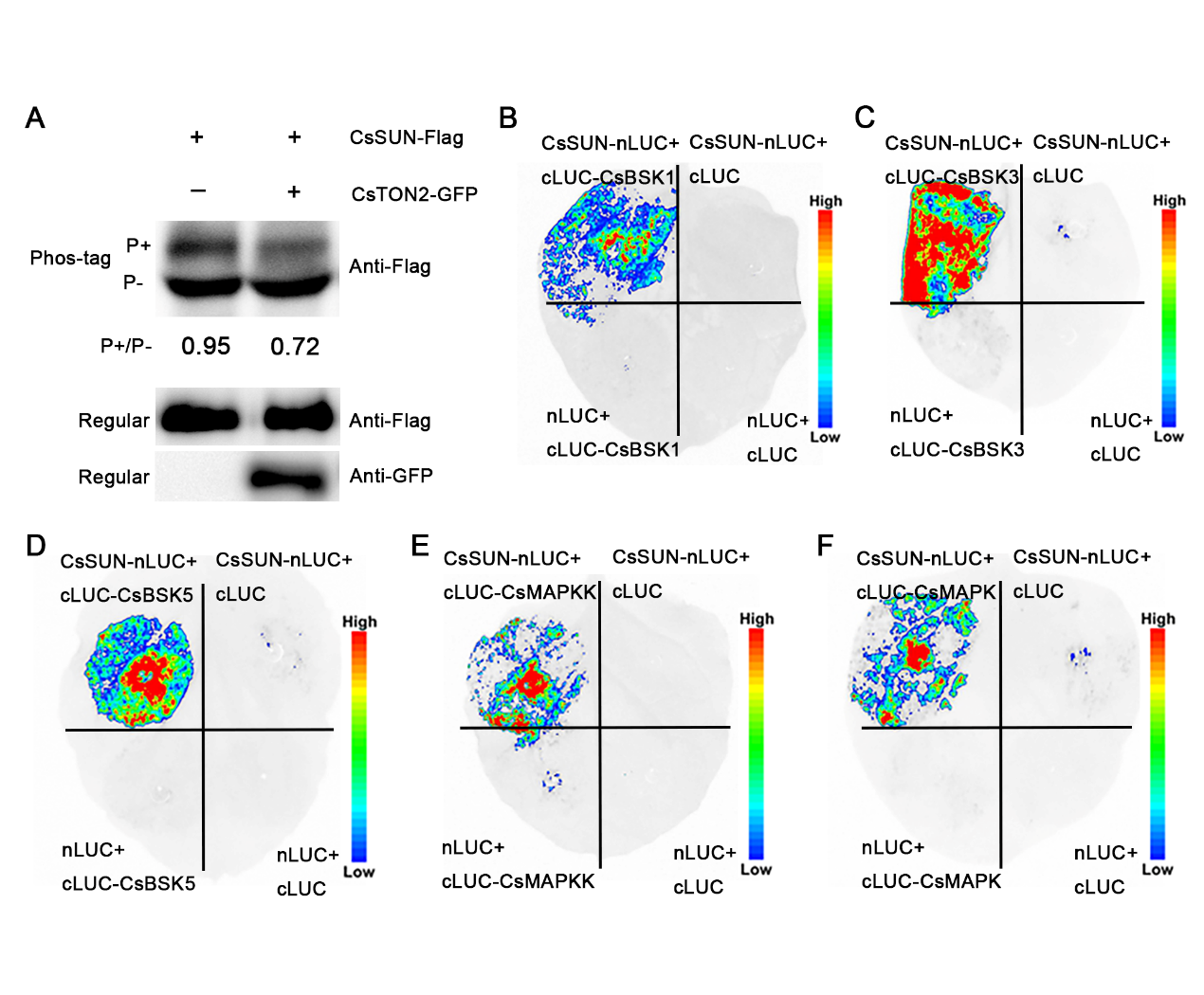


**Figure S7. CsTON2 regulated CsSUN phosphorylation and CsSUN-interacting kinase proteins.**

(A) Immunoblotting analysis showing that CsTON2 dephosphorylates CsSUN in *N.benthamiana*. The protein of CsSUN-Flag was separated in Phos-tag gel. The intensity of the bands was measured with the Image J software and calculated the ratio of P+/P−. Total proteins in the reaction system were detected by the anti-Flag antibody. CsTON2-GFP was detected by anti-GFP antibody. (B-F) The interaction of CsSUN with CsBSK1 (B), CsSBSK3 (C), CsBSK5(D), CsMAPKK (E) and CsMAPK (F) revealed by firefly luciferase complementation imaging assay in *N.benthamiana* leaves. The luciferase proteins were fused to the N-terminus of CsSUN, and the C-terminus of CsBSK1, CsBSK3, CsBSK5, CsMAPKK and CsMAPK, respectively.


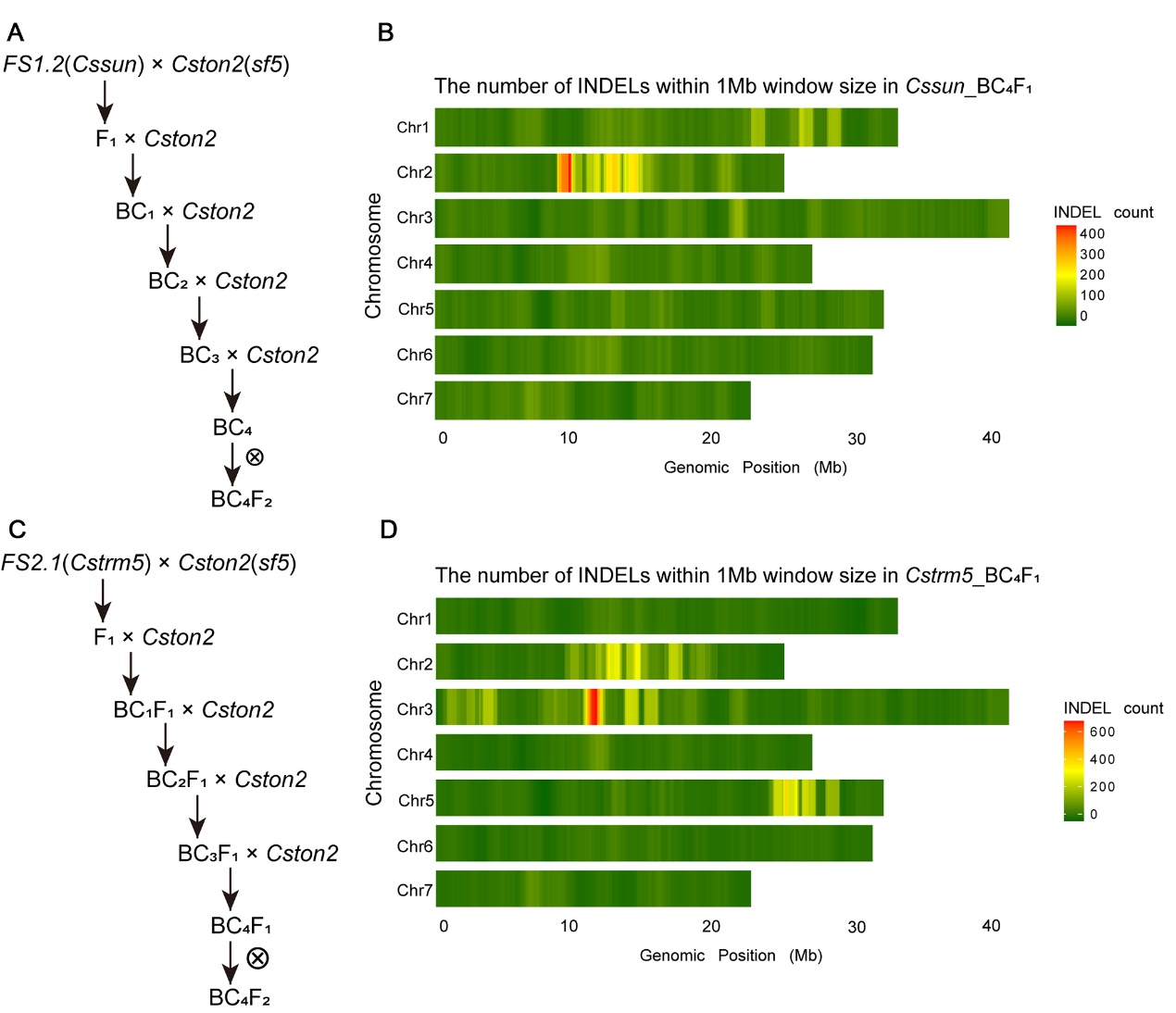


**Figure S8. Construction of *Cssun* and *Cstrm5* NILs.**

(A) Schematic diagram of the generation of *Cssun* NILs. (B) Whole-genome sequencing results of *Cssun*_BC_4_F_1_. (C) Schematic diagram of the generation of *Cstrm5* NILs. (D) Whole-genome sequencing results of *Cstrm5*_BC_4_F_1_, using the Chinese Long 9930 v3 genome as reference. The color key (green to red) represents the number of InDels.


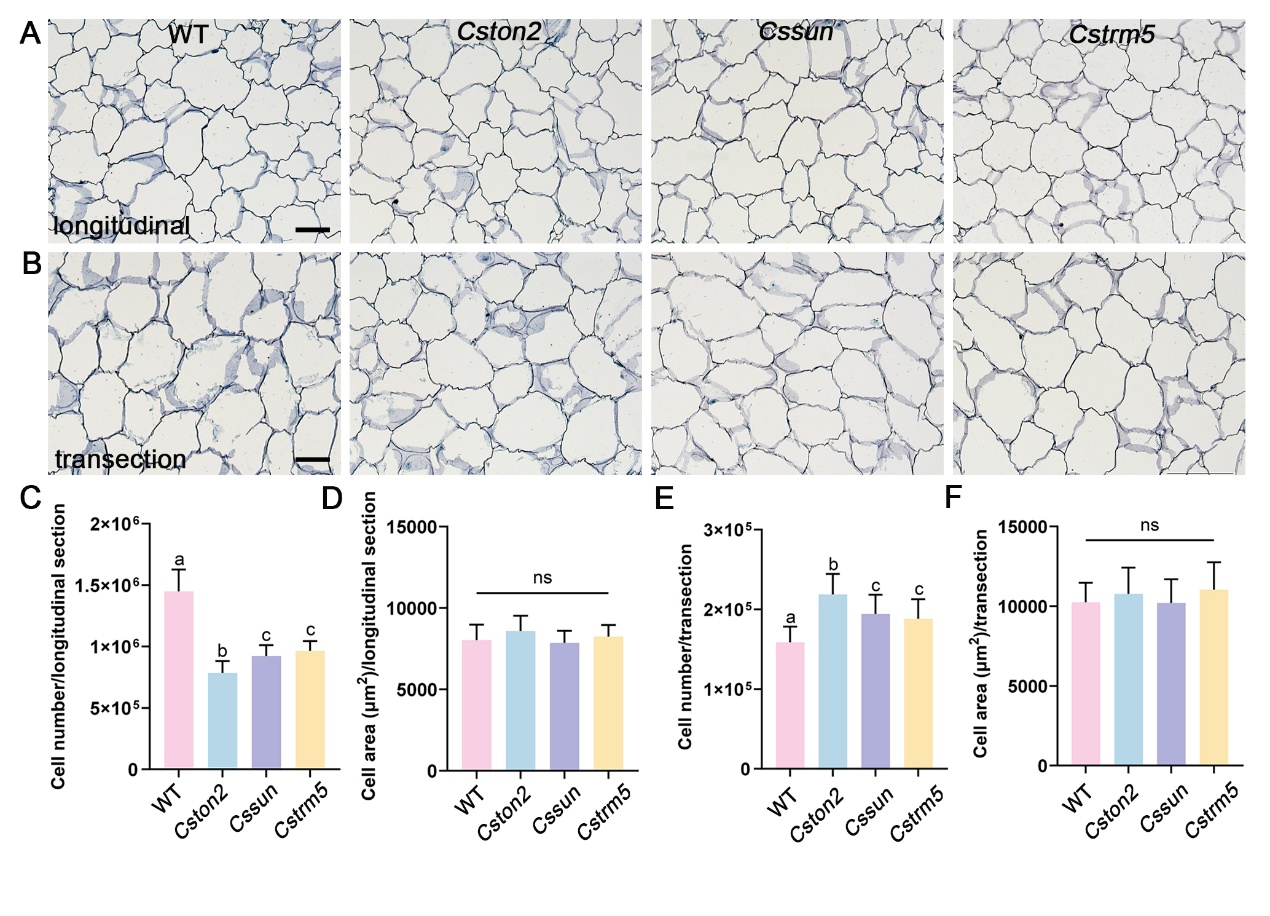
**Figure S9. CsTON2, CsTRM5 and CsSUN regulate fruit shape by influencing cell division.**

(A-B) Longitudinal sections (A) and transections (B) in the mesocarp at 10 DAA of WT, *Cston2*, *Cssun*, and *Cstrm5*, Scale bars = 100μm. (C-F) Cell number and cell size statistical of the fruit pericarp in longitudinal sections (C-D) and transections (E-F) of WT, *Cston2, Cssun*, and *Cstrm5*. Data are the means ± SD, n ≥ 18. Different letters represent significant differences by one-way ANOVA with Tukey’s post hoc test (*P* < 0.05). ns, no significant difference.
