# Supplementary material for: The Microtubule‐Associated Protein CsTON2 Interacts With CsTRM5 and CsSUN to Regulate Fruit Shape Development in Cucumber
Source: Plant Biotechnol J. 2025 Dec 29;24(4):2725–41. doi: 10.1111/pbi.70519 (PMC13140649; doi:10.1111/pbi.70519)
Supplement: Supplementary file 2 — Table S1: Inheritance analysis of fruit shape in sf5 mutant in cucumber. Table S2: Genes information used in this study. [file PBI-24-2725-s003.docx]

**Table S1** Inheritance analysis of fruit shape in *sf5* mutant in cucumber.

| **Population** | **Long fruit phenotype** | **Short fruit phenotype** | **Segregation ratio** | **Expected ratio** |
| --- | --- | --- | --- | --- |
| P1 (*sf5*) | 0 | 12 |  |  |
| P2 (WT) | 14 | 0 |  |  |
| F1 (P1 × P2) | 15 |  |  |  |
| F1 (P2 × P1) | 19 |  |  |  |
| F2 | 188 | 65 | 2.8923:1 | 3:1 |

**Table S2** Genes information used in this study.

| **Gene name** | **Species** | **Accession** |
| --- | --- | --- |
| *AtTON2* | *Arabidopsis thaliana* | AT5G18580 |
| *SolyTON2* | *Solanum lycopersicum L* | Soly01g067500 |
| *MtTON2* | *Medicago truncatula* | *Medtr4g119230* |
| *GmTON2* | *Glycine max* | Glyma.07G250200 |
| *CmoTON2* | *Cucurbita moschata* | CmoCh06G014760 |
| *CmTON2* | *Cucumis melo* | MELO3C006623P1 |
| *CsTON2* | *Cucumis sativus* | CsaV3_3G014300 |
| *ZmTON2-1* | *Zea mays L* | Zm00001d024857 |
| *ZmTON2-2* | *Zea mays L* | Zm00001d010862 |
| *OsTON2* | *Oryza sativa* L | LOC_Os05g05710 |
| *CsSUN* | *Cucumis sativus* | CsaV3_1G039870 |
| *CsTRM5* | *Cucumis sativus* | CsaV3_2G013800 |
| *CsBSK1* | *Cucumis sativus* | CsaGy6G010390 |
| *CsBSK3* | *Cucumis sativus* | CsaGy5G002680 |
| *CsBSK5* | *Cucumis sativus* | CsaGy6G014000 |
| *CsMAPKK* | *Cucumis sativus* | CsaGy2G000730 |
| *CsMAPK* | *Cucumis sativus* | CsaGy1G021930 |
| *CsUbiquitin* | *Cucumis sativus* | CsaV3_5G031430 |
